# Evidence of memory from brain data

**DOI:** 10.1093/jlb/lsaa078

**Published:** 2020-12-18

**Authors:** Emily R D Murphy, Jesse Rissman

**Affiliations:** University of California Hastings College of the Law, 200 McAllister Street, San Francisco, CA 94131; Psychology and Psychiatry & Biobehavioral Sciences, University of California, Los Angeles

**Keywords:** brain, court, evidence, fMRI, machine learning, memory detection

## Abstract

Much courtroom evidence relies on assessing witness memory. Recent advances in brain imaging analysis techniques offer new information about the nature of autobiographical memory and introduce the potential for brain-based memory detection. In particular, the use of powerful machine-learning algorithms reveals the limits of technological capacities to detect true memories and contributes to existing psychological understanding that all memory is potentially flawed. This article first provides the conceptual foundation for brain-based memory detection as evidence. It then comprehensively reviews the state of the art in brain-based memory detection research before establishing a framework for admissibility of brain-based memory detection evidence in the courtroom and considering whether and how such use would be consistent with notions of justice. The central question that this interdisciplinary analysis presents is: if the science is sophisticated enough to demonstrate that accurate, veridical memory detection is limited by biological, rather than technological, constraints, what should that understanding mean for broader legal conceptions of how memory is traditionally assessed and relied upon in legal proceedings? Ultimately, we argue that courtroom admissibility is presently a misdirected pursuit, though there is still much to be gained from advancing our understanding of the biology of human memory.

## I. INTRODUCTION

In 2008, Aditi Sharma was convicted by an Indian court of killing her fiancé, Udit Bharati.^1^ The court relied in part on evidence derived from the so-called Brain Electrical Oscillations Signature (BEOS) test.^2^ To take this test, Sharma sat alone in a room wearing a skullcap with protruding wires that measured brain activity under her scalp. She listened to a series of statements detailing some aspects of the murder that were based on the investigators’ understanding. Throughout the test, she said not a word. But when she heard statements such as ‘I had an affair with Udit’, ‘I got arsenic from the shop’, ‘I called Udit’, ‘I gave him the sweets mixed with arsenic’, and ‘The sweets killed Udit’, the computer analyzing her brain activity purported to detect ‘experiential knowledge’ in her brain.^3^ In the subsequent 6 months, the same lab provided evidence in the murder convictions of two more people.^4^

Recent advances in peer-reviewed research on memory and its detection present an important question for evidence law: what would it mean to be able to detect the contents of a person’s memory? If a brain-based approach were scientifically reliable, would it be admitted as courtroom evidence? Admissibility in court and the persuasiveness (or prejudicial effect) of evidence is often the focus of legal analysis of the new brain science technology.^5^ Indeed, some memory detection researchers perceive courtroom admissibility to be the *sine qua non* for forensic applications of memory detection technology.^6^

Both the inventors of BEOS and some US-based researchers insist that their brain-based memory detection technologies are not ‘lie detection’ and should not be painted with the same brush of unreliability and thus inadmissibility.^7^ Others have claimed that memory detection technology will soon be admissible as evidence.^8^ But ‘admissibility’ is not an inherent quality of novel technology, but rather a complicated legal, factual, and scientific question in a particular case. Because admissibility is a recurrent focal point for some researchers and advocates, and because brain-based memory detection technology has been admitted in some jurisdictions,^9^ we offer a framework for the assessment of evidentiary use of brain-based memory detection in trial proceedings. The primary focus for this analysis will be on memories of witnesses and suspects as the most likely targets of forensic applications.^10^

The admissibility of memory detection evidence is a complex legal question not just because it requires careful judicial gatekeeping of complex scientific evidence, but also because it directly concerns one of the core ‘testimonial capacities’ of a witness, and thus directly bears on the jury’s task of assessing witness credibility. Part I introduces the conceptual framework for memory detection research being translated to forensic practice. Part II provides an overview of the current state of the technology, with further details for scientific readers published elsewhere.^11^ Part III sets up the framework for assessing courtroom admissibility, establishing the nature of the factual and legal issues raised by the current technological capabilities and scientific understanding. Memory detection may also implicate individual constitutional and compulsory process rights, limiting or enabling its use as courtroom evidence.^12^ Part IV then analyzes the potential objections to memory detection as courtroom evidence, starting with those that are surmountable challenges before moving onto stronger objections and normative considerations. Finally, Part V concludes by considering the use of the technology outside of the adversarial context. The question that this analysis presents is: if the science is in fact sophisticated enough to demonstrate that accurate, veridical memory detection is limited by biological, rather than technological, constraints, what should that understanding mean for broader legal conceptions of how memory is traditionally assessed and relied upon in legal proceedings? Ultimately, we argue that courtroom admissibility is presently a misdirected pursuit, though there is still much to be gained from advancing our understanding of the biology of human memory.

## II. MEMORY DETECTION: THEORY TO FORENSIC PRACTICE

Human memory plays a critical role in many different aspects of law and legal proceedings, not the least of which is witness testimony. For example, decades of scientific research into the nature of memory have recently supported significant structural legal developments, such as changes in pretrial motion practice and jury instructions about evaluating eyewitness identification testimony.^13^ Thus, periodic assessment of what we know about memory and can do in terms of its detection is important for legal and interdisciplinary scholars. Parts I and II synthesize several lines of research centering around the question: is it possible to detect the presence (or absence) of a specific memory? The answer depends both on advances in measurement (in technology and behavioral test design) and also on the better understanding of the biological (including psychological) nature and limitations of human memory—an ongoing subject of basic science research.

Significant technological advances have been made in neuroimaging since this literature was last comprehensively reviewed for an interdisciplinary audience,^14^ and a key question now is whether remaining limitations are fundamentally technological or biological problems. Technological problems are of the kind that permits researchers and commentators to say ‘in the future, we may be able to…’ based on advances in technology. Biological problems, in contrast, may be true boundary conditions on what type of detection or characterization may be possible. As technology advances, what seemed like biological problems may yield to advanced scrutiny.

### II.A. What Is (and Is Not) Memory Detection?

What do we mean when we refer to ‘memory detection’? Let us first distinguish memory detection from lie detection, or ‘truth verification’.^15^ The existence (or absence) of a memory trace^16^ could theoretically be detected regardless of whether the subject is affirmatively misrepresenting or concealing that information.^17^ This article evaluates work aimed at detecting the presence or absence of recognition of some sort of stimuli, rather than deception *per se*.

Researcher Peter Rosenfeld and colleagues explain that a canonical version of the ‘guilty knowledge test’ used in most memory detection protocols ‘actually does not claim or aim to detect lies; it is instead aimed at detecting whether or not a suspect recognizes information ordinarily known only by guilty perpetrators and, of course, enforcement authorities’.^18^ That is, present forms of memory detection require the human designing the test to know something about the ground truth of interest, and obtain or design stimuli and testing protocols to determine whether the subject also has that knowledge. Presently, no brain-based memory detection technology functions as one might imagine ‘mind-reading’ to work, via uncued reconstruction of the subjective contents of a subject’s memory.^19^

In theory—and consistent with lived experience—our brains are generally able to distinguish autobiographical memories (such as having personally witnessed or participated in an event) from other sources of event knowledge (such as knowing details of people’s lives that we have read or heard about, or knowing the mere fact that an event occurred at a given time and place).^20^ That is, we know the stories of our own lives and can generally tell the difference between our own lives and the events and information in the world around us. Of course, our brains are not perfect at this task, and normal people experience spontaneous memory errors (such as the powerful experience of déjà vu, the subjective feeling of having previously lived through a current, novel experience)^21^ as well as imagined or suggested memory errors (sometimes referred to as ‘source confusions’ because we misattribute the source of our event knowledge).^22^

Exactly how the brain distinguishes autobiographical memories from other memories, and the base rates of inaccuracies or distortions, is the subject of ongoing memory research. For example, very recent research suggests that different kinds of episodic memories—varying in their degree of autobiographical content—may be neurobiologically distinguishable.^23^ A neurobiological distinction would not be surprising. Rather, it would be expected that distinct biological mechanisms underlie how these different types of memories are subjectively experienced. These findings, explored further below in Part II, may have significant import for assumptions about the ecological validity to be assumed from laboratory-based memory creation and detection, even using mock crime scenarios, and real-world memory creation and detection. Each of these characteristics contributes to a technique’s false positive and false negative rate, which are critical measures for any test used to classify an outcome as present/absent.

As with methods of ‘truth verification’, a potential forensic appeal of memory detection is based on an essentialization—the assumption that certain brain activity is more automatic, less under conscious control, and less subject to fabrication, reinterpretation, or concealment than subjective reports or even physiological measurements of the body such as skin conductance, heart rate, breathing rate, and even eye movements.^24^ One way this assumption has been tested is through more rigorous attempts to quantify how vulnerable different kinds of memory detection are to countermeasures—deliberately applied behavioral or cognitive strategies for ‘beating the test’ or manipulating the test results. But active countermeasures are only one form of potential distortion; others come from the innate imperfections of human memory.^25^ How these ‘sins’ such as transience, absent-mindedness, misattribution, and subsequent misinformation constrain memory detection is a question now squarely raised by the most recent research on functional magnetic resonance imaging (fMRI)-based memory detection, discussed in Part II.

Some proponents of forensic memory detection tend to approach the problem as one of technological limitations, and indeed the literature reviewed below is organized primarily by methodology. More complex technology (fMRI and machine-learning data analysis) has enabled major advances in memory detection and scientific knowledge about the nature of memory. But it has also brought us closer to asking whether the constraints on memory detection may be ‘biological problems’ as well as technological problems. That is, which limitations on memory detection come from the nature of memory itself?

Most obviously, for many aspects of our experiences, a memory beyond a brief sensory trace never gets formed. As an *a priori* theoretical matter, it will not be possible to detect a memory that was never formed because the information or stimulus was never attended to. But as an empirical matter, it is extremely difficult to test for the true absence of something, and thus it would be extremely difficult for a negative result from a memory detection test to be interpreted as the true absence of a memory and thus a true lack of personal experience. Conversely, lots of things we do not pay attention to may nonetheless get stored into memory at some level. These unattended stimuli are less likely to be consciously remembered and will sometimes be completely forgotten, but other times will influence behavior in subtle ways.^26^

A second challenge is that, even if a memory were formed, it may be degraded or forgotten to a degree that it is no longer detectable. This boundary condition is at least theoretically easier to experimentally investigate—a memory trace could be detected at time one, and then be absent when probed at a later time. But the mere act of testing for the memory could, itself, modify or strengthen the memory, thus preventing ‘normal’ degradation and forgetting.^27^ The reactivation of a memory through questioning or testing procedures could strengthen a degraded recognition, and/or a subject’s confidence in their recall.^28^ How such influences impact the detectability of a memory will have a critical impact upon the marginal utility of brain-based memory detection versus testimony of compliant, but perhaps forgetful, witnesses.

### II.B. What Kinds of Memories Are to Be Detected?

The next important concept is to define precisely what kind of memories we are most interested in detecting and what kind of memories various tests actually detect or could detect. For laypersons without a psychology background, this may seem obvious: we would like to detect true/veridical memories, rather than false, inaccurate, or distorted memories. False memories in legal proceedings are an important area of psychological study with many legal implications; detailed consideration of which has been undertaken elsewhere.^29^ For our purposes, it is important to hone in on what kinds of memories forensic applications would likely be most interested in detecting: objectively true, autobiographical memories.

Broadly speaking, autobiographical memories can be semantic (facts about yourself such ask knowing the places you have lived and the names of your family members and friends) or episodic (recollection of events that happened to you at a specific time and place).^30^ Both are a type of ‘explicit’ or ‘declarative’ memory: generally speaking, those that can be consciously recalled and described verbally. These may be contrasted with ‘implicit’ memories, where current behavior is influenced by prior learning in a non-conscious matter, such as the procedural skill of riding a bike. Although some researchers in this field have limited the categories of forensic interest to episodic memories, there may be types of semantic autobiographical memories of forensic interest such as names of criminal associates whom one has never personally met, targets of attack one has only been told, or one’s true name or place of birth.^31^

The taxonomy just described is a drastic simplification of an active and ongoing area of research—just ‘how’ memory should be classified and operationalized—and one that does not seem to be in active dialogue with memory detection researchers. A recent review described a categorization of ‘personal semantic’ (PS) memory—knowledge of one’s past—as an intermediate form of memory between semantic and episodic memory.^32^ PS memory has been ‘assumed to be a form of semantic memory, [thus] formal studies of PS have been rare and PS is not well integrated into existing models of semantic memory and knowledge’.^33^ The authors describe four ways in which PS memory has been operationalized as autobiographical facts, self-knowledge, repeated events, and autobiographically significant concepts. Depending upon how PS memory is operationalized, in different studies it appears neurobiologically more similar to general semantic memory or episodic memory, meaning that tasks activating each show similar patterns of neural activation on functional brain imaging. Though a sophisticated field of research with a voluminous literature, the difficulties in coming to a consensus about the taxonomic nature of memory are in part because memory is extremely difficult to study.^34^

One final thing must be said about a type of autobiographical memory that is not the subject of memory detection studies, despite being extremely relevant as courtroom evidence: memories, or beliefs, about one’s past mental states. Memories of ‘events that happened’ are the subject of brain-based memory detection research. This is not the case for memories of ‘past subjective mental state’, notwithstanding the concept’s obvious relevance to legal issues of *mens rea*. Of course, a past *mens rea* could potentially be inferred from the detection of a memory of a specific event or item. For instance, if brain-based memory detection could establish that a defendant had actually seen bullets in a gun’s chamber, such evidence might weigh against the defendant’s claim that he believed the gun was unloaded. But direct detection of past mental states, even if considered as a subtype of autobiographical memory, has not been attempted, and nor is it clear how it could be. Although a single recent study suggested that brain imaging could distinguish between present, active mental states of legal relevance (knowing vs. reckless, to the extent those legal concepts can be operationalized for behavioral study)^35^, the ‘time travel’ problem will not likely be resolved by increasing technological sophistication.^36^

## III. STATE OF THE ART IN BRAIN-BASED MEMORY DETECTION

With this conceptual framework in mind, brain-based memory detection has biological and technological axes to examine.^37^ The biological axis considers and explores how memory actually works in the brain. The technological axis is concerned with the tools used to explore that biology. Advances in understanding on one axis reciprocally inform the other.

Organization of this part by technological method allows understanding of technological improvements and approaches to the challenges discussed above, which helps set the stage for understanding where limitations are biological, rather than technological, in nature. We start with electroencephalography (EEG)-based technology, which uses electrodes to detect electrical brain activity through a subject’s scalp. We then discuss fMRI, which uses powerful magnetic fields to non-invasively detect correlates of brain activity. Those readers who are interested in the high-level summary and analysis may skip ahead to Part II.C.

### III.A. E‌EG-Based Technology

There is now a substantial body of research using EEG-based techniques to detect memories, reporting impressive results on accuracy of detection, often exceeding 90 per cent. EEG-based technologies are deployed in various commercial efforts^38^ and are in forensic use internationally.^39^ This section will first review the basic technique and theory behind EEG-based memory detection, and then report on recent findings and remaining limitations on forensic use.^40^ Notably, a small handful of research groups have dominated the EEG-based memory detection research efforts. The large bulk of peer-reviewed publications comes from the lab of Peter Rosenfeld et al at Northwestern University.^41^

EEG-based memory detection protocols measure electrical brain activity from dozens of small electrodes placed on the scalp as a subject is presented with a series of stimuli—typically words or pictures shown on a computer screen, or sounds played through headphones or speakers. EEG-based memory detection protocols vary, and subtle variations in those protocols are the subject of intense research because small changes in testing protocol have large impacts on results.

The fundamental concept relies on a test framework exploiting the brain’s different responses to personally meaningful versus non-meaningful stimuli, called the ‘orienting response.’^42^ The logic of EEG-based memory detection protocols is that the person administering the EEG can measure the subject’s brain responses to different stimuli and use them to discriminate between meaningful or non-meaningful information. In this context, a stimulus that is ‘meaningful’ is one that would be salient or significant only to someone with prior knowledge or experience with that stimulus, such that its ‘meaningfulness’ is at least partially based on recognition memory. Thus, the nature of the information presented in the stimuli—which must be carefully selected beforehand by the test administrator—is the most important methodological factor in EEG-based memory detection.

Nearly all EEG-based memory detection protocols attempt to detect a characteristic waveform of electrical activity evoked by meaningful/familiar/salient stimuli, and in most research this waveform is the P300 ‘event related potential’, or ERP.^43^ An ERP is a particular spike or dip in brain voltage activity in response to a discrete event (such as a bright light or loud noise). The P300 is a positive ERP occurring between 300 and 1000 milliseconds after the onset of events that are both infrequent and meaningful, with a maximal effect typically observed for those electrodes situated over the parietal lobe.^44^ A shorthand way to think of what the P300 represents is a response to stimuli that are ‘rare, recognized, and meaningful’.^45^

As mentioned above, EEG-based detection relies on the comparison of brain responses between different categories of stimuli. The majority of protocols is based on the concealed information test (CIT; also known as the ‘guilty knowledge test’) and use three types of stimuli: (i) infrequent ‘probe’ stimuli with information relevant to the event of interest that would only be recognized by someone with prior knowledge (that is, meaningful to some people but meaningless to others); (ii) infrequent ‘target’ stimuli that subjects are explicitly instructed to monitor for and respond to with a unique button-press—these are stimuli that the subject is expected to know, recognize, or be familiar with (meaningful to everyone); and (iii) frequent ‘irrelevant’ stimuli, of no relevance to the event of interest nor of any particular importance to the subject (meaningless to everyone). For all subjects, the target stimuli should elicit a greater P300 response amplitude than the irrelevant stimuli, providing an internal check that the procedure is working and the data are of sufficient quality. Most critically, for ‘guilty’ examinees, probe stimuli should elicit a P300 similar in magnitude to that observed for target stimuli, indicating that their brain ‘knows’ the probe stimuli are not irrelevant. Conversely, the brains of ‘innocent’ examinees should show little no P300 response to the probes, such that this waveform will look more similar to that observed for irrelevant stimuli than for target stimuli.^46^

An important point to appreciate is that for EEG-based memory detection, subtle variations in methods can substantially affect the results. The past several years of research have largely been dedicated to these methodological challenges. One methodological constraint of much of EEG-based memory detection deserves particular attention, which is that most studies report results on ‘blocks’ of trials for a given condition, rather than a single trial. Generally speaking, the reason for this is that EEG data are extremely noisy, such that a meaningful ‘signal’ can only be pulled out from background ‘noise’ when averaging responses over several trials of a given condition.^47^ As such, to detect the ERP, researchers typically average the EEG samples of repeated presentations of the same stimulus, or of several stimuli in the same category. That is, relatively smooth-looking graphs of P300 responses should be understood to be averages of multiple repeated trials. Indeed, typically several hundred presentations of test items are required for reliable recording of the P300 ERP.^48^

Researchers have been studying the use of the P300 ERP to detect ‘concealed information’ since the 1980. The first P300-based protocol was based on the CIT.^49^ Eventually, the standard CIT task was determined to be vulnerable to countermeasures,^50^ and Rosenfeld and colleagues developed a new countermeasure-resistant variant, which they dubbed the ‘Complex Trial Protocol’ (CTP). The CTP is designed to enhance the difficulty of the standard CIT and reduce the likelihood of success that a subject could secretly designate a category of ‘irrelevant’ probes as ‘relevant’, thus reducing the difference in P300 amplitudes between ‘probes’ and secretly relevant ‘irrelevants’ for guilty subjects—essentially beating the test by creating their own false positives.^51^ The reasoning behind the countermeasure vulnerability of the older three-stimulus protocol is that when covert target and probe stimuli competed for attention resources, the P300 response is reduced. This proposed mechanism highlights a particular analytic weakness of needing to use an internal comparison between different categories of stimuli to detect whether one subset is recognized or recalled.^52^

As discussed, much of the recent research in EEG-based memory detection has focused in recent years on subtle changes in protocol design. Those that are particularly significant for purposes of assessing forensic capacity and potential admissibility are methodological developments focused on more sophisticated countermeasure vulnerability, issues of ecological validity, and, most recently, the application of pattern recognition techniques to analyze EEG data. The highlights of those areas of research, and consideration of the outstanding limitations for forensic use, are briefly discussed below.

#### III.A.1. Sophisticated countermeasure vulnerability

*Alternative mental actions as countermeasures*. Several studies have shown that subtle physical countermeasures, such as instructing participate to wiggle a toe or finger during the presentation of certain stimuli (as a means to increase their salience), can be highly effective at preventing experimenters from obtaining an accurate assessment of concealed knowledge.^53^ But countermeasures could be even more covert. Mental countermeasures such as silently ‘saying’ one’s own name in response to different ‘irrelevant’ stimuli would be nearly impossible to detect through even the closest observation of a test subject.^54^

But does using mental countermeasures slow down task performance, such that countermeasure use could be detected? Early work with the CTP found that slower reaction times indicated countermeasure use.^55^ This telltale slowing seems to work only when subjects execute the covert countermeasure separately from a required ‘I saw it’ motor response. If participants are trained to execute both at the same time, this ‘lumping’ strategy can eliminate the ability to use reaction time as an indication of countermeasure use, though the P300 ERP signal can still detect more than 80 per cent of the ‘guilty’ test subjects who were trained to use this strategy (note that this is still a significant reduction from reported detection thresholds exceeding 90 per cent).^56^ That is, there may be countermeasures to countermeasure detection strategies, possibly able to characterize mendacious subjects, though without high degrees of certainty.

*Voluntary suppression of memory.* Can someone beat a memory detection test by voluntarily suppressing their memories? Two papers in 2013 and 2015 reported that the P300 signal for episodic information could be voluntarily suppressed.^57^ But it may be possible to design the task in a slightly easier way so as to protect against this countermeasure.^58^ Moreover, it may not be possible to suppress responses to probes targeting semantic memories, such as the knowledge of one’s true name.^59^ At present, the effect of countermeasures on well-constructed CTP applications appears to be modest and perhaps available only to highly trained subjects. Nevertheless, a complete understanding of potential countermeasure vulnerability is critical to accurate forensic application of any P300-based memory detection protocol, especially research that can differentiate the effects of task demands from mechanisms of countermeasure deployment.

#### III.A.2. Ecological validity

Ecological validity is the extent to which laboratory conditions and results translate to those in the real world. A canonical memory detection study with low ecological validity is having a subject study a list of random words, then asking her to respond whether or not certain words were on that list, while attempting to conceal the actual studied list with false responses. Studying a list of random words is not a task that resembles real world or forensically relevant events, and it is unwise to generalize from this kind of task to a forensic task that involves presenting a subject with a list of words that may be relevant to a crime being investigated. Alternatively, studies aiming for greater ecological validity may engage their subjects in mock crime scenarios, such as ‘stealing’ a particular item and then attempting to conceal their ‘crime’ from the examiners. These types of studies began with the earliest efforts at EEG-based memory detection.^60^ But with all studies using volunteers, the genuine motivation to engage in and then conceal truly criminal or antisocial actions cannot be replicated, as of course all laboratory ‘thefts’ are designed and sanctioned by researchers.

*Use of real-world scenarios and detecting autobiographical memory in individuals.* A fundamental question for forensic application of memory detection technology is the extent to which lab-created memories—even those with more realistic mnemonic content than a memorized word list, such as a mock crime—are similar to or meaningfully different from real-world memories. There are many reasons such memories could be different, including the amount of attention a subject is allocating to lab-based, instructed tasks such as stealing a document versus going about normal routines of the day when something unexpected happens.

A recent study has tried to address the detectability of incidentally acquired, real-world memories.^61^ Meixner and Rosenfeld had subjects wear a small video camera for 4 hours, as they went about their normal daily routine. The next day, subjects returned to the lab and, while hooked up to the EEG, were shown words associated with activities they experienced the previous day (such as ‘grocery store’), as well as irrelevant words of the same category but not relating to the subject’s personal activities (such as ‘movie theater’ or ‘mall’).^62^ The authors reported that the EEG data could be used to perfectly discriminate between the 12 ‘knowledgeable’ subjects who viewed words related to their personal activities and the 12 ‘non-knowledgeable’ subjects who simply viewed irrelevant items. Notably, this was the first P300 study to examine whether autobiographical memory could be detected at an individual level, since most psychological studies of autobiographical memory use group averaged data, which is not helpful for forensic purposes. But, it still presents the question of whether comparisons between subjects are necessary to make a decision about an individual’s ‘knowledgeable’ status. More importantly, the study design does not quite get at the question of whether real-life memories are different, at some important neural level, from lab-created memories (which most studies investigate)—it simply suggests that this particular tool can be used to discriminate between subjects who had or did not have discrete real-life experiences.

*Innocent-but-informed participants.* Other work focused on assessing the ecological validity of a P300-based CIT demonstrated that prior knowledge of crime details has an effect on detection rates, illustrating potential risks of using probes that may have become known to an innocent subject (such as someone who received instructions to steal a ‘ring’ but did not go through with the crime).^63^ These ‘innocent-but-informed’ participants were ‘essentially indistinguishable’ from those who actually committed the mock crime: ‘Simple knowledge of probe items was sufficient to induce a high rate of false positives in innocent participants’.^64^ Rosenfeld and colleagues counsel that crime details must remain secret, known only to police, perpetrators, eyewitnesses, and victims. But how feasible is it for investigators to know for certain that the probed details are, indeed, secret? This may be easier said than done, and it is not always possible to know the degree to which critical details about the event in question have been inadvertently disclosed. Further relevant to forensic application, the studies investigating variables reviewed in this section have not distinguished between witnesses (who may be innocent-but-informed) and participants (‘guilty’ subjects), such that variables such as time delays and quality of encoding are poorly understood in applications of detection of witness (rather than suspect) memory.

*Time delay between event and test.* In lab studies using mock crimes, participants are often tested immediately or a few days after the incident. In the real world, interrogation about an event may come weeks, months, or even years later. One study attempting to quantify the impact of a delay in testing asked students to ‘steal’ an exam copy from a professor’s mailbox.^65^ Some students were tested using a P300-based CTP procedure immediately after the theft, whereas others returned to the lab a month later. Researchers found no difference in detection efficiency. This is an encouraging result, but more evidence is needed. The test only used a single probe item (the stolen exam), and—as with other mock crime scenarios—subjects were explicitly instructed to engage in the theft, likely heightening the salience of the central detail. What is presently unknown is how P300-based detection fares over time for peripheral crime details, which may be less robustly encoded, but more likely to be uniquely known only to a perpetrator, and thus useful to minimize a false positive result.

*Quality of encoding and incidentally acquired information.* Not all information is equally well remembered. This is, of course, an important feature of human memory, as it would be highly inefficient for a lawyer to remember where she parked her car two Tuesdays ago equally as well as the legal standard for summary judgment. How well information is remembered has substantial implications for how well it may be detected. Yet behavioral tests of people’s memory for incidental details of real-world experiences show that sometimes surprisingly little is retained.^66^ Rosenfeld’s research group acknowledges that sensitivities in their P300-based tests are less with incidentally acquired information than with well-rehearsed information.^67^ Strategies to improve the sensitivity of detection of incidentally acquired information under investigation include providing feedback to focus a participant’s attention on the probe^68^, using an additional ERP component,^69^ and combining separately administered testing with the CTP.^70^ This is a critically important problem for memory detection, as incident-relevant details important for determining guilt may be only ‘incidentally encoded’, particularly under conditions of stress. Is this a problem that can be addressed with technical advances, such as those explored by Rosenfeld et al? Or will the technical improvements simply reveal the outer boundaries of conditions under which subtle but critical details may be recalled and detected, given that they were not central to the incident but may be necessary for avoiding a false positive? It is well known that stimuli that are unattended during learning are often only weakly remembered, or not remembered at all, when assessed on later recognition test.^71^

Existing research has focused on how the use of countermeasures by a guilty subject may lead to missed detection—a false negative because of countermeasures. What has not been firmly established is how often a guilty subject will fail to show a P300 response to a probe stimulus for another reason, such as the fact that he may not have encoded the particular details of a weapon used, because of intoxication, darkness, mental illness, impulsivity, or stress—attributes that may be more prevalent in a criminal defendant population than in a research population.^72^

*Quality of retrieval environment.* A final concern for ecological validity is the effect of stress or other contextual factors on memory ‘retrieval’ as well as encoding.^73^ Most P300 studies using volunteer subjects—even those who participate in a mock crime—cannot fully replicate the stress of a real-world, high-stakes memory probe of a suspect (or witness). Although studies generally suggest that stress has a negative effect on episodic memory retrieval,^74^ the effect of real-world stress on EEG-based memory detection is not adequately studied.

### III.B. fMRI Based Technology

Functional magnetic resonance imaging is a safe, non-invasive, and widely used research and clinical tool. The details of how it works have been reviewed extensively elsewhere in the legal literature, so only a high-level reorientation is provided here for the unfamiliar reader.^75^ Functional magnetic resonance imaging uses powerful magnetic fields and precisely tuned radio waves to detect small differences in blood oxygenation that serve as a proxy for neural activity. When a population of neurons becomes active, the brain’s vascular system quickly delivers a supply of richly oxygenated blood to replenish the metabolic needs of those neurons. At present, some fMRI scanners are capable of collecting a snapshot of the blood oxygenation level-dependent signal across the entire human brain every 1 or 2 seconds at reasonably high spatial resolution (ie the images are comprised of 2–3 mm cubes called ‘voxels’).^76^ Subjects must lie with their head very still in a large, loud tube, but can perform behavioral tasks during scanning by looking at visual stimuli presented via a projection screen (or a virtual reality headset)^77^, listening to audio stimuli over headphones, and/or making responses using a keypad or button-box.

Two attributes in particular distinguish fMRI-based memory-detection from EEG-based memory detection. The first is technological access to more and different biological sources: fMRI can provide data from the entire brain. Multiple, interconnected brain regions are involved in forming and retrieving memories. One physiological limitation with EEG-based memory detection technologies is that EEG predominantly measures cortical electrical responses—the outer covering of the brain closest to the scalp—but cannot measure signals from ‘deeper’ brain structures such as the hippocampus. Although fMRI offers slower temporal resolution than EEG (that is, brain activity levels are sample on the order of seconds in fMRI, instead of milliseconds in EEG), fMRI offers substantially greater spatial resolution across the entire brain.

The second attribute is technological with respect to analysis capability: the most advanced and interesting fMRI studies of memory detection leverage the power of massive amounts of data obtained from brain scans to actually assess complex network connections and use machine-learning algorithms to recognize subtle patterns in networks, rather than activation in local areas.^78^ These analytic techniques, combined with experimental paradigms and research questions directly aimed at assessment of ecological validity concerns, represent significant advancement in the technological aspects of memory detection. These methodological advances permit assessment of where biological constraints may lie that put ultimate boundaries on forensic use.

#### III.B.1. Early fMRI work on true and false memories

Early fMRI work examining neural correlates of true and false memories reported dissociations—that is, unique differences—between brain areas activated in response to test stimuli in a recognition task asking subjects to remember which stimuli they had previously studied.^79^ A recent review of this literature identified as a ‘ubiquitous’ finding the ‘considerable overlap in the neural networks mediating both true and false memories’ for recognition responses to items that share the same ‘gist’ as items that were actually studied during encoding.^80^ Many studies also find differences—notably, increased activity in regions related to sensory processing for true as compared to false retrieval, leading to the ‘sensory reactivation hypothesis’ that true memories are associated with the bringing back to mine of more sensory and perceptual details than false memories.^81^ Overall, empirical support for the intuitively appealing sensory reactivation hypothesis is mixed, and other dissociations such as different patterns of activity in the medial temporal lobe and prefrontal cortices have been reported.^82^ Other work testing episodic memory retrieval in an fMRI scanner a week after viewing a narrative documentary movie reported that the coactivations of certain brain areas were greater when subjects responded correctly to factually accurate statements about the movie, but such coactivations did not differ between responses to inaccurate statements about the movie.^83^ Collectively, this work provided some suggestion that activation patterns could differentiate between true and false recognitions, based on distinct memory processes, though no clear potential for diagnostic assessment of true or false memories emerged. That is, there is no particular ‘spot’ in the brain that serves as a litmus test for whether a memory is true or false.

#### III.B.2. Newer fMRI methods: advanced techniques reveal the biological limitations of memory detection

A limitation of the classic fMRI analysis paradigms is that memories do not exist in discrete regions of the brain. Memories are encoded and stored in networks of brain regions.^84^ Moreover, EEG studies using event-related potentials and fMRI studies using classic ‘univariate’ contrasts of brain activity are only equipped to gage the relative level of activation across different regions of brain, and the level of activation provides only rudimentary information about one’s memory state. But newer fMRI analysis methods can make use of the massive amounts of data to assess complex network connections and use machine-learning algorithms to recognize subtle activity patterns in networks, rather than activation in local areas.

This is a substantially more powerful way to analyze complex data, and the remainder of this review will focus on fMRI studies that employ such methods. These methods offer the best hope for reliable forensic memory detection, the greatest insights into the biological limitations of memory detection, and the subtlest challenges for a fact finder to assess the credibility of the techniques used to make claims about the presence or absence of a memory of interest.

### III.C. Multi-Voxel Pattern Analysis and Machine-Learning Classifiers

Classic fMRI analysis looks at chunks of the brain: clusters of voxels and regions of interest. By analyzing each voxel separately, this ‘univariate’ analysis approach ignores the rich information that is encoded in the spatial topography of the distributed activation patterns—that is, it spotlights a particular area, but misses patterns in the broader network of brain activity. But the brain is highly interconnected, and not organized as a highly localized series of components.

A newer ‘multivariate’ technique, multi-voxel pattern analysis (MVPA), tries to exploit the information that is represented in the distributed patterns throughout a brain region or even across the entire brain. It is a more sensitive method of analysis because it is more adept at detecting distributed networks of processing. MVPA techniques use machine-learning algorithms to train classifiers on data patterns from test subjects. The classifier learns the distributed ‘neural signatures’ that differentiate unique mental states or behavioral conditions. Once adequately trained, the classifier is then tested on new fMRI data (that it has not been trained on) to determine whether it can accurately classify the condition of a subject’s brain on a given trial based solely on brain data information.^85^ Simply put, there is more informational content in fMRI activity ‘patterns’ than is typically detected with conventional fMRI analyses. The accuracy with which the classifier can discriminate trials from Condition A and Condition B gives a quantitative assessment of how reliably two putatively distinct mental states are differentiated by their brain activity patterns.

MVPA has enabled significant advances in memory detection research. In 2010, the first paper to apply an MVPA approach to memory detection in fMRI ‘evaluated whether individuals’ subjective memory experiences, as well as their veridical experiential history, can be decoded from distributed fMRI activity patterns evoked in response to individual stimuli’.^86^ Before entering the scanner, participants studied 200 faces for 4 seconds each. In the scanner about an hour later, they were presented with the 200 studied faces interspersed with 200 unstudied faces and pressed a button to indicate whether or not they recognized a given face. Participants were accurate about 70 per cent of the time, giving researchers the ability to examine their brain activity both when they were correct and when their memory failed them. The classifier was first trained to differentiate brain patterns responding to old faces that subjects correctly recognized from brain patterns responding to new faces that they correctly judged to be novel—both situations in which the subjective experience and objective reality of the response were identical. The classifier performed well above chance, with a mean classification accuracy of 83 per cent, and rising to 95 per cent if only the classifier’s ‘most confident’ guesses were considered. But since subjects did not perform perfectly—sometimes misidentifying new faces as having been previously seen, or rejecting old faces as novel—the classifier could also be tested on the ‘subjective’ mnemonic experience. In those scenarios, the classifier performed relatively poorly—near chance—when applied to detect the ‘true’ experiential history of a given stimulus on those trials for which participants made memory errors. That is, the classifier proved to be very good at decoding a participant’s ‘subjective’ memory state, but not nearly as good at detecting the true, veridical, ‘objective’ experiential history of a given stimulus. Subjective recognition, of course, can be susceptible to memory interference—resulting in commonly experienced, but false, memories.^87^

Three other aspects of this study deserve separate mention, as they are particularly relevant to assessing the forensic capabilities and limitations of fMRI/MVPA-based memory detection. First, though many stimuli were shown to subjects in order to train the classifier, once trained it could be applied to single trials—that is, a face shown just one time provided sufficient neural information for the classifier to make a categorization decision.^88^ This is a significant advantage over EEG-based memory detection paradigms that require multiple presentations to detect an event-related potential, as well as ‘classic’ univariate fMRI analyses that assess averages of trials across conditions. A subsequent study confirmed that the MVPA classifier could decode the memory status of an individual retrieval trial, and attempted to assess the vulnerability of this single-trial assessment to countermeasures by instructing participants who had studied a set of faces to attempt to conceal their true memory state.^89^ Participants were instructed to feign the subjective experience of novelty for any faces they actually recognized and to feign the experience of recognition for any faces they did not recognize (eg by recalling someone that the novel face reminded them of). Using only this easy-to-implement countermeasure strategy, participants were able to prevent the experimenters from accurately differentiating brain responses to studied versus novel faces, with the mean classification accuracy dropping to chance level.^90^ That is, although MVPA classification of fMRI data can enable single-trial memory assessment, it may still be vulnerable to simple mental countermeasures.^91^

Second, the researchers specifically considered whether the same classifier would work across subjects by training it on data from some individuals, but then testing it on data from others. This would be an important feature of any technology to be forensically applied, but depends on an assumption of some unknown degree of consistency across the brains of different people. The classifier worked well across individuals, ‘suggesting high across-participant consistency in memory-related activation patterns’.^92^ This is a significant result in that it suggests that, biologically, different people’s brains may be similar enough in how they process memories for technological solutions to be somewhat standardized.

Third, recall that the ‘classifier’ is not a person making a judgment call—it is a machine-learning algorithm, in this case based on regularized logistic regression^93^—assessing patterns of neural data. What the 2010 study suggests is that memories that feel true but are objectively false may have neural signatures quite similar to memories that feel true and are true.^94^ If that finding holds, it has substantial implications for the ability to detect true memories and avoid detection of false memories.

### III.D. Advances in Experimental Paradigms with MVPA: Real-World Life Tracking, Single-Trial Detection, and Boundary Conditions Revealed

Lab-created memories such as studying a series of faces may be relatively impoverished, from a neural data perspective, as compared to real-life memories that have potential for context and meaning. It is possible that where MVPA-based detection met limits for lab-created memories (in particular, the inability to distinguish objectively false but subjectively experienced memories, and vulnerability to simple countermeasures), additional information available to the classifier from a richer memory set may make real-life memories more distinguishable. This enrichment of the memory experience, and delays between the experience and time of test, also address concerns about ecological validity of research for potential forensic application.^95^

To address this, Rissman and colleagues had participants wear a necklace camera for a three-week period while going about their daily lives before returning a week later to be scanned while making memory judgments about sequences of photos from their own life or from others’ lives.^96^ After viewing a short sequence of four photographs depicting one event, participants made a self/other judgment and then indicated how strong their experience of recollection or familiarity was for photos judged to be from their own life, or their degree of certainty about photos judged to be from someone else’s life.

Behaviorally, participants were quite good at this task, successfully distinguishing which events were from their own life or someone else’s life on around 80 per cent of trials, with the remaining 20 per cent of trials split between incorrect or ‘unsure’ responses. Indeed, participants performed so well that there was not sufficient data to assess whether the classifier could distinguish objectively true from objectively false (but subjectively experienced as true) memories. That is, the 2016 study was not helpful in distinguishing false from true memories, because there was not enough false memory data to work with. This is perhaps a side effect of the fact that real-life memories are richer than lab-created memories like pictures of faces or words, and perhaps less susceptible to spontaneous false-memory effects. Nevertheless, an fMRI classifier trained to distinguish one’s own life events from others’ life events performed extremely well, succeeding at classifying the self/other status of individual events 91 per cent of the time on average, with no subject’s classification performance below 80 per cent.^97^ When the classifier was required to distinguish between trials where participants reported recollecting specific details from those in which they reported only familiarity for the event, it still performed well above chance, with mean accuracy of 72 per cent.

One feature of regularized logistic regression machine-learning classifiers is the ability to create ‘importance maps’, which permit researchers to assess which voxels (and thus, networks of brain areas) are important to the classifier making the decision. Based on these importance maps, self/other classifier distinctions relied on brain areas associated with mnemonic evidence accumulation and decision processes, whereas recollection/familiarity distinctions showed a very different pattern, involving brain regions associated with the retrieval of contextual details about an event.

This study design also addressed the issue of retention interval—that is, the amount of time that elapses between the formation of a memory and the probing of that memory in the magnetic resonance imaging (MRI) scanner. The classifier that was tested on memories that occurred 1–2 weeks before the scan performed just as well as a classifier tested on memories collected 3–4 weeks before the scan, at the beginning of the time participants started wearing the cameras. This suggests that recent memories are not necessarily easier to decode than more remote memories.^98^ However, this study only assessed memories that were at most 1 month old. One earlier fMRI study that also used wearable cameras compared memories that were 5 months old to those that were only 36-hours old and found that the older memories evoked a somewhat different profile of brain activity, including less activation of the hippocampus and surrounding medial temporal lobe structures that commonly associated with episodic recollection.^99^ Given that some, if not most, potential forensic applications will involve the need to probe memories that are months or years old, more research is needed to determine how reliably classifier-based memory detection will work on older memories, as well as whether classifiers are capable of estimating the age of a probed memory.

As with the previous study, researchers also confirmed that a classifier trained on some individuals will perform well when tested on different participants, suggesting that the underlying brain activity patterns are fairly consistent across subjects. Moreover, the researchers attempted to directly address the question of whether lab-based memories are different from autobiographical memories by using the classifier from the 2010 faces memory study, on brain data from the 2016 self/other life photograph study, and *vice versa*. In both situations, the classifier still succeeded at predicting a subject’s mnemonic judgment over two-thirds of the time, well above chance.^100^

Although the 2016 Rissman et al study could not answer whether real-world true and false memories differed in neural activation patterns, a 2015 fMRI study from a separate research group used MVPA classification methods and a mock crime scenario similar to the EEG-based CIT to approach the issue.^101^ One group of subjects (guilty intention) planned a realistic mock crime (a theft of money and a CD with important study information), but did not actually commit it. Another group (guilty action) planned and executed the ‘crime’. And a third group (informed innocent) was informed of half of the relevant details in a neutral context, but did not engage in any planning intention. Subjects were scanned during a CIT behavioral task. The MVPA analyses showed that although it was possible to reliably determine whether or not individual subjects possessed knowledge of crime-relevant details, the classifier was far less accurate (and indeed not significantly better than chance) at discriminating between the subjects in the three groups.^102^ In other words, the classifier could not tell whether the presence of crime-related memories had been obtained by way of crime execution, crime planning, or merely reading about the crime-relevant details. Thus, much like the comparable EEG study discussed above,^103^ even the informational richness of whole-brain fMRI brain activity patterns may be insufficient to prevent the risk of false positive identifications of innocent-but-informed individuals.

Of course, researching real-life memories in a laboratory setting is methodologically complex; what about the fact that looking at pictures is itself an autobiographical experience? How do ‘laboratory memories’ really diverge from ‘real-world memories’, and can a classifier tell the difference?^104^ Recent work by the Rissman group manipulated the self/other task (using photographs from necklace-mounted cameras) by permitting subjects to preview some portion of photographs a day before scanning.^105^ Essentially, the study asked whether there is a detectable neural difference between the experience of viewing a photograph and the experience of actually living a particular experience. This is indeed what was found; the classification analyses revealed a dissociation between the diagnostic power of each of two different large-scale brain networks. Specifically, activity patterns within the ‘autobiographical memory network’ were significantly more diagnostic than those within the ‘laboratory-based network’ as to whether photographs depicted one’s own personal experience, regardless of whether they had been viewed before scanning. In contrast, activity patterns within the laboratory-based memory network were significantly more diagnostic than those within the autobiographical memory network as to whether photographs had been previewed, regardless of whether they were from the participant’s own life. This dissociation provides some evidence for separate neural processes for retrieval of firsthand experience versus secondhand knowledge—a finding that has significant implications for how, in a forensic context, stimuli are selected and whether or not they can or should be previewed to subjects.

### III.E. So Where Is Brain-Based Memory Detection Now?

The foregoing covered a lot of science, even while skipping over critical technical details such as receiver-operating characteristic curves, parametric versus non-parametric statistical testing, and a heavy amount of advanced math, not to mention the wide range of technical details about individual scanner parameters, data processing, and data analysis packages.^106^ Without wanting to indicate that these details are unimportant—because in fact their accessibility is critically important should this technology ever be introduced in court, some of which will be discussed below—we provide here the high-level summary of the state of the art of brain-based memory detection technology in the context of what it might mean for legal applications.

The most advanced brain-based memory detection work leverages algorithmic classification of rich networks of brain activity. This work is substantially advancing the basic science research in memory studies—including helping determine what kinds of subtly different cognitive processes have distinct neural substrates. This work also leverages real-life experiences, is able to assess subjective mnemonic status at a single-trial, individual level, and can use a classifier trained on data other than from a subject of interest, indicating some conservation of neural networks for particular memory tasks across people. In short, the ‘technological’ aspects of the work are so sophisticated that we may start to be confident that MVPA-based memory detection research is beginning to reveal the ‘biological’ limitations of memory detection.

This is not to say that further technological developments could not reveal clear biological distinctions between true or false or modified memories. Indeed, the understanding of which machine-learning classifiers perform better when applied to different brain areas/networks is rapidly evolving,^107^ and may give further insight into the underlying nature of autobiographical memory. But at this point, the technology confirms biological processes of memory that are congruent with our best understanding of memory encoding and retrieval processes consistent with years of psychological research—fundamentally, that memory is an inherently reconstructive process.

At present, the most salient limitations are these: that even with sophisticated technology able to detect distinctions in different types of autobiographical or episodic memory processes, there may be no way for a brain scanner and machine-learning algorithm to (i) distinguish between a false, but subjectively believed memory and an objectively true memory, (ii) detect, on a single-trial level, the deployment of simple mental countermeasures, and (iii) distinguish between someone who has knowledge of or even intention for, but did not participate in, a particular event. If these findings reflect ‘biological’ truths rather than ‘technological*’* limitations of detection, there is a serious boundary condition on the utility of brain-based memory detection to contribute to accurate fact-finding, if the issue is what really happened, rather than what a subject thinks or believes.

Moreover, in all research conducted thus far, researchers had perfect access to the veridical truth about the world—the memory of which is assessed in the scanner—such as by controlling the mock crime scenario or selecting the photographs from the wearable cameras. What remains unknown is how such technologies would work when investigators have varying degrees of uncertainty about which stimuli should match a person’s recollection or trigger recognition. For example, would photos of a terrorist training camp trigger recognition or recollection if they were from a vantage point that a suspect had never seen?^108^ Would a years-old photo of an associate’s face, with a different hairstyle, eyewear, and countenance, elicit recognition? Were details known only to a crime participant and investigators, such as the paisley-patterned couch the victim was found on, really encoded by the perpetrator?^109^ What if someone had committed a past burglary, but not the burglary under investigation, though a probe item such as a burglary tool elicited an essentially false positive recognition?^110^ Or might memories be more reliably detected for crimes like trafficking and financial crimes, where a perpetrator has repeated exposures to a particular place, face, or documents? Also unknown, yet critical to determining the ‘accuracy’ (as in positive and negative predictive value) of any diagnostic test, are real-world base rates of inaccurate memories, such as false positives (memory present and detected, but not actually experienced) and false negatives (event experienced, but memory not present or detected). These ‘false memory’ base rates might even vary between different types of factual scenarios in which memory detection would want to be forensically applied, discussed below.

The residual uncertainty about accuracy in real-world applications is a familiar gap between research science and forensic science. Because of the inherently reconstructive nature of human memory,^111^ it is not a gap likely to be completely bridged even by accessing the brain doing the reconstruction. Nevertheless, advances in the ability to use brain-based measures to detect real-life memories have potential value for deployment in legal and social contexts. But that value needs to be carefully assessed to be appropriately deployed in context.

## IV. THE DOCTRINAL PATH TOWARDS COURTROOM USE: RELEVANCE, RELIABILITY, AND CREDIBILITY ASSESSMENT

The law relies on memory in myriad ways.^112^ Some are about fact-finding: most obviously, memory is often the primary—or only—source of information about past events that have legal relevance. Others are assumptions about how memory works that are entrenched in doctrine and legal standards.^113^ A memory detection technique that could confirm the presence or true absence of memories of disputed facts would have a massive impact on many types of legal proceedings, well beyond trial testimony.

Evidence law is of course focused on filtering the testimonial and other evidence that reaches a jury. Because ‘admissibility’ is not an inherent property of a particular technology, but rather a mixed question of facts, law, and science dependent on the purpose for which the evidence is offered, this Part lays the doctrinal path toward courtroom use of brain-based memory detection. The questions raised by potential courtroom applications are slightly different from the issues raised by technological and biological limitations described above, chiefly along the dimension that in different courtroom applications, varying degrees of knowledge about ground truths and certainty in assessment may be acceptable, depending on who is offering the memory detection evidence and for what purpose. Ultimately, this part and the following argue that courtroom admissibility should not be the focus of memory detection technology development, for reasons both pragmatic and doctrinal.

### IV.A. Judicial Gatekeeping: Relevance, Reliability, and ‘Fit’ of Brain-Based Memory Detection

Next, we map the steps toward memory detection evidence being admitted in court: relevance, reliability, and the ‘fit’ for the offered purpose. The admissibility of memory detection evidence will be subject to judicial gatekeeping because it will be the subject of expert testimony.^114^ As a preliminary matter, admissible expert evidence must be relevant. Relevance is a low threshold; as long as the memory detection evidence tends to make a fact in issue even more or less probable, it would be considered relevant.^115^ A memory need not be disputed to be in issue, but if testimony about a memory were not in doubt^116^ and not pertaining to a central fact in issue in the case, it is more likely that a judge would exclude the cumulative evidence on the grounds of wasting time.^117^

For memory detection evidence to be relevant, we must consider what kind of memory it is capable of detecting, and thus what legal scenarios it is applicable to. As described above, brain-based memory detection can potentially detect autobiographical memories of an act or an event, or semantic memory such as unique factual knowledge that proves identity. What was not investigated in the work described above is the ability of brain-based technologies to detect past intent or past mental state; this is presently not possible, and may never be—though this is an issue going to inherent reliability, rather than relevance *per se*.^118^ But the current state of knowledge means that memory detection evidence is probably not relevant in cases where the factual issue is proving mental state or intent, which is almost certainly more frequently disputed than the act or event of ‘what exactly happened’ or ‘who dun it’.^119^

Still, memory detection evidence is likely relevant in civil cases where the disputed issue is ‘what happened?’—that is, what are the objective facts about past events—perhaps encompassing many torts, as well as employment harassment or discrimination cases. Memory detection of prior knowledge of a patent or prior art may be relevant in claims of willful infringement or inequitable conduct.^120^ In criminal cases, the relevance and utility of memory detection may be more limited than proponents might think because memory detection cannot directly assess *mens rea*. If memory detection can only access memory of autobiographical events or semantic knowledge, it would be most useful in criminal cases for: corroboration or impeachment purposes, in the same way that some states permit polygraphs for such purposes;^121^ eyewitness identification, where someone is misidentified, and the witness has an incorrect memory that can be proven to recognize someone else; alibis lacking corroboration, where a defendant says ‘it wasn’t me!’, but cannot offer other proof that s/he was elsewhere except with experiential memory from the same time period; and possibly in cases where a defendant claims a confession was coerced, in that they admitted to something they did not do, and would have no experiential memory of (though this would require confidence that the absence of a memory was truly evidence of the absence of the experience). And even in these hypotheticals where it might be relevant, there are of course reliability issues with whether any brain-based memory detection technology can ever distinguish between memories that are objectively true and those that are false, but subjectively believed to be true.

What about reliability? Memory detection evidence would be offered via the opinion of an expert, where reliability is assessed under *Daubert* and its progeny, Federal Rule of Evidence 702, or state law equivalents, discussed next. But imagine for a moment a world where memory detection evidence was offered directly by a party, because the technology had been around long enough that an expert’s specialized knowledge was no longer ‘helpful’ to a jury.^122^ Direct evidence of memory detection would pose an analytical challenge fascinating to hearsay scholars.^123^ If such evidence were scientifically valid and reliable, nearly to the point of infallible assessment of a veridical, objective truth, it could be considered free from one of the major sources of unreliability that normally infects hearsay—that of faulty memory that could not be revealed by cross-examination and juror assessment of witness credibility. Arguably, if memory detection were capable of validating a veridical, objective truth, it also addresses the other worried-about sources of unreliability: misperception, sincerity, and narration.^124^ A perfect version of memory detection could solve all of those problems, eliminating reliability concerns that underscore the hearsay prohibition, but squarely presenting the question of what would be left for the jury. As a thought experiment, memory detection as a ‘hearsay solution’ raises interesting but familiar questions about the conflicting values, in addition to a search for accuracy, about our commitment to a jury system. These we address below in Part IV.

In terms of mechanics of assessing reliability, memory detection evidence would be offered as the opinion of an expert, probably the person who designed and administered the test. An expert (or two) would have to testify on two issues: whether the test is reliable and valid for the use to which it is being put (closely tied to the relevance considerations above), and if so, whether it was reliably employed in a given case to produce an accurate result. Expert testimony would be a necessary vehicle for memory detection test results given the scientific and technological expertise necessary to understand the psychological theory, choose appropriate stimuli, and perform the technological assessment of brain-based memory detection. Potential hearsay concerns about the machine’s direct output are for now set aside^125^; an expert opinion can be based on evidence that would otherwise be inadmissible as hearsay.^126^ In the federal system and many states, the *Daubert* trilogy of cases and Federal Rule of Evidence 702 (or state analogs) govern the method by which judges must do an initial, gatekeeping assessment of the admissibility of an expert’s testimony.

*Daubert* analyses of brain-based deception (lie) detection techniques abound in the literature, nearly all agreeing that such applications fail to clear the admissibility threshold for lack of understood error rates.^127^ Courts that have considered brain-based deception detection technology have agreed, finding it inadmissible at present.^128^ But some commentators argue that the distinctions between tests that purport to detect deception and those that simply detect recognition should ‘radically affect’ the admissibility analysis under *Daubert*.^129^ This is analytically incorrect, for reasons explained *infra* Part III.B.1. To the contrary, the *Daubert* or *Frye* standards should at present continue to exclude expert testimony opining that brain-based memory detection proves the presence (or absence) of a particular memory in a given subject.^130^ With respect to ‘general acceptance’ standards, leading researchers and many recent papers in the fMRI-based memory detection space caution that because of the limitations in existing studies and the nature of the findings about inherent limitations on memory detection, much more research is needed before forensic applications are pursued.^131^ There is far from a ‘general acceptance’ of these technologies for forensic application at present.

Reliability will vary as a function of the type of memory being assessed. Memory detection will work best in situations where a subject has a repeated experience resulting in a sturdy, non-fragile memory. Based on what we know about the nature of memory, it is virtually certain that there would be different base rates in memory inaccuracies depending on the type of memory and a number of factors about its encoding and retrieval. The phenomenon of memory contamination or false memories is well studied, but incomplete as an epidemiological field of study. For example, we now know that even just ‘imagining an event that might have occurred in someone’s past can increase confidence or believe that the event actually occurred, lead individuals to claim that they performed actions that they in fact only imagined or result in the production of specific and detailed false memories of events that never actually happened’.^132^ We also know that normal people, describing non-traumatic life events over successive interviews, show high degrees of variability in their autobiographical memory.^133^ What we do not really know are the relevant base rates—that is, how often false or inaccurate memories happen in day-to-day life.^134^ Not only are error rates of memory detection technologies unknown, but these base rates of different kinds of inaccuracies in memory are also unknown for real-world applications and may never be ascertained.

Nevertheless, validation studies will accumulate, and error rates will be proposed. Would they be sufficient to cross the admissibility threshold? *Daubert* is focused on reliability through validation.^135^ But this standard is vulnerable to misinterpretation and thus insufficient scrutiny at the admissibility stage because validation studies for forensic memory detection would be inherently limited for at least two reasons.

First, they are necessarily conducted under idealized conditions with respect to the person being examined. In the research context, investigators can control for individual subject variables that may impact reliability such as mental health status, head injury status, intoxication status, stress level, and demographic features such as level of education.^136^ Second, as with forensic DNA evaluation or other forensic machine evidence in the form of statistical estimates and predictive scores, validation studies are potentially an incomplete method of ensuring accuracy.^137^ Real validation is feasible in laboratory procedures that show that a measured physical quantity, such as a concentration, consistently lies within an acceptable range of error relative to the true concentration. But where the ‘true concentration’ cannot be known—because investigators do not know the ground truth, or because individual memories are inherently reconstructed—there is no underlying ‘true’ value that exists.^138^ This problem veers into the epistemic—how can we ever know what is true? But it is precisely because brain-based memory detection’s appeal depends upon an assumption of essentialization of lived experience to empirical truth^139^ that this type of evidence deserves epistemological scrutiny as part of its validation.

That is, imperfect validation studies may be enough under existing law to get brain-based memory detection studies admitted, but they may be insufficient to permit the jury to decide, in a nuanced way, whether to believe and how much weight to assign to them. Even if *Daubert-Frye* prerequisites for brain-based memory detection were arguably met, it is important to recognize that a *Daubert*-*Frye* analysis does not, on its own, provide sufficient information for a fact finder to perform an adequate ‘credibility’ analysis, discussed *infra* Part III.B and Part IV, though it may supply the veneer of such scrutiny.^140^

Finally, the outcome of the reliability gatekeeping scrutiny is framed by which party is offering the technology, and for what purpose—the issue of ‘fit’. Because criminal defendants have a constitutional right to compulsory process, such that evidentiary rules that would bar admission must sometimes yield,^141^ a defendant offering brain-based memory detection in support of his defense may be able to offer brain-based memory detection that is perhaps less reliable than what the prosecution would have to put forward. When the Supreme Court considered lie detection technology in *United States v. Scheffer,* the Court held that a defendant does not have a right to present polygraph evidence, but reached this majority opinion because Justice Thomas’ fifth vote in concurrence was based on his conception of the jury as lie detector.^142^ In a dissent largely driven by due process concerns—given that Scheffer wanted to offer his polygraph results in his own defense—Justice Stevens wrote that ‘evidence that tends to establish either a consciousness of guilt or a consciousness of innocence may be of assistance to the jury in making such [credibility] determinations’, and argued that juries could follow the instructions of a trial judge concerning the credibility of an expert witness. The polygraph’s bid for admissibility failed in *Scheffer* because it was ‘too’ unreliable to overcome constitutional due process concerns.^143^ But if brain-based memory detection technology can do better than the polygraph in terms of accuracy and reliability,^144^ it might be admissible if offered by a criminal defendant.

In contrast, memory detection technology may effectively be held to a higher standard of reliability if offered by the state in a criminal prosecution. Yet other constitutional due process concerns arise when the state is proffering. Although a brain-examined witness must consent to have the state physically examine their brain activity via skull cap electrodes or by lying perfectly still in a MRI machine, memory detection technology does not necessarily require any overt response from the subject, spoken or otherwise.^145^ Completely unresolved is the constitutional question of whether the output of a memory detection device would be considered simply physical evidence, or ‘testimonial’ for purposes of the Confrontation Clause of the 6th Amendment.^146^ This is a philosophical and doctrinal problem beyond the scope of this article, but suffice it to say that a defendant might raise a Confrontation Clause challenge to memory detection evidence of a state witness unavailable for cross-examination at trial.

It is possible to imagine that a highly accurate, highly reliable brain-based memory detection device could clear the judicial gatekeeping hurdle of admissibility. It would necessarily have to be a good ‘fit’, in terms of its relevance and reliability, in the factual and legal context in which it is offered. At this stage of our analysis, it seems those contexts would be rather limited, and thus a project of ‘admissible’ memory detection technology might not be pragmatic or efficient.^147^ Moreover, constitutional due process and compulsory process concerns frame the ultimate question. What remains to be considered is that brain-based memory detection is essentially evidence of a witness’s credibility, and what this means for its courtroom use.

### IV.B. Credibility Assessment: Brain-Based Memory Detection Is Evidence about Credibility, and Biological Limitations Mean that Brain-Based Memory Detection Tests Are Really Best at Assessing Witness Sincerity—Just Like Lie Detectors

Even if a gatekeeping judge could be satisfied that brain-based memory detection evidence survives the doctrinally required scrutiny for reliability and relevance, the task for the jury is not a binary one of accepting or rejecting the evidence. The jury must assign weight to the conclusions of the expert. For brain-based memory detection, this task would implicate two layers of credibility analysis, complicating the task of separately and appropriately weighing of each layer.

Credibility, in the context of evidence law, means simply whether a source of information is worthy of being believed^148^—not merely whether a witness is lying.^149^ That is, credibility assessment, properly understood, is not limited to just the ‘hearsay danger’ of insincerity.^150^ The other ‘testimonial capacities’ of ambiguity, memory loss, and misperception are tested in human witnesses by oath, physical confrontation, and cross-examination. Were brain-based memory detection admitted in court, via an expert witness, it should be recognized to be ‘double-credibility dependent proof’.^151^ That is, a fact finder could be led to draw the wrong inference about the content of a person’s memory, and ultimately a relevant fact, because of potential infirmities of both sources: the memory itself (that is, witness credibility), and undetected ‘black box’ dangers leading to imprecise or ambiguous outputs and incorrect inferences from memory detection technology.^152^

This ‘double credibility’ analysis is not sufficiently scrutinized by existing *Daubert* and *Frye* reliability requirements for expert methods.^153^ Furthermore, the ‘credibility’—not just the sincerity—of human witnesses is canonically the province of the jury. Outsourcing credibility assessment to a brain-based memory detection technology and expert witness reporting raises serious, but familiar, issues about the role of a jury system.^154^ These potential objections to the use of brain-based memory detection as courtroom evidence are addressed in Part IV.

This section argues that in many applied contexts, memory detection is probably not practically distinguishable from lie detection—and thus is subject to the same objections regarding the role of the jury as the ultimate assessor of credibility. The most advanced scientific and technological work in memory detection reviewed above presently suggests that no machine, no matter how sophisticated, could detect a false but subjectively believed memory—that is, an inaccurate memory that someone truly believes they experienced.^155^ This is not of small consequence in a forensic context; the phenomenon of memory contamination or false memories is well studied.^156^ As discussed above, what we do not really know are the relevant base rates—that is, how often false or inaccurate memories happen in day-to-day life.^157^

The key point is that, depending upon the situation at hand, brain-based memory detection may offer little to no probative value in assessing the ‘accuracy’ of a witness’s memory due to the biological properties of memory itself. If that finding reflects a scientific truth—that brain activity for false and true memories cannot be reliably distinguished—then the utility of brain-based memory detection remains even more firmly in the zone of assessing the testimonial capacity of witness ‘sincerity’—based on a match or discrepancy between the results of the test and any other witness statements—and subject to the same objections as lie detector tests that it impermissibly impinges upon the role of the jury by bolstering or impeaching a witness’s credibility.

Not all commentators agree; John Meixner distinguishes ‘neuroscience-based credibility assessment’ from ‘evidence that directly assesses whether a witness is telling the truth’,^158^  ^159^ arguing that only the latter should be understood to invade the province of the jury, relying principally on language from a plurality in *Scheffer*.^160^ In terms of the jury’s role and the law of evidence, this is a distinction without a difference. Credibility assessment is a broader task than detecting active deception or mendacity. Juries are meant to be lie detectors, but they are equally charged with assessing the amount of weight to give to each piece of admitted evidence. They do so by assessing four testimonial capacities of a witness: perception, memory, narration, and sincerity, and by considering a piece of evidence in context with all others. Thus, a technology that detects memories impinges just as much on the province of the jury to assess ‘memory’ as an element of credibility as does a lie detection technology impinges on the assessment of ‘sincerity’*.* The point here is simply that memory detection technologies are, from a standpoint of permissible (and thus admissible) evidence, similarly situated to lie detection technologies. The issue of whether the role of credibility assessment is or should be exclusively one for the jury is discussed below in Part IV.A.1, as a weaker objection to the evidentiary use of memory detection technology.

## V. OBJECTIONS TO COURTROOM USE OF BRAIN-BASED MEMORY DETECTION

John Henry Wigmore was optimistic that the courts would embrace something like a perfect memory detection device and did not limit the utility of such a device to deception or mendacity: ‘But where *are* these practical psychological tests, which will detect specifically the memory-failure and the lie on the witness-stand? … If there is ever devised a psychological test for the valuation of witnesses, the law will run to meet it…. Whenever the Psychologist is really ready for the Courts, the Courts are ready for him’.^161^ A memory detection device, better than a psychologist, seems to be the epitome of what he had in mind.

Given the preference in the rules of evidence for admissibility of relevant evidence, we have mapped the path of legal and factual issues leading toward courtroom admissibility of brain-based memory detection. We turn now to remaining objections as to why this technology should not be used in court. These are organized from weak objections and surmountable challenges, some of which memory detection has in common with other types of scientific or expert evidence; to strong objections inherent to the technology and task; to normative and philosophical objections that encompass familiar concerns about our commitment to trial by jury (and its alternatives), the value of dignity in and outside of the courtroom, and the role of memory in the nature of personhood. Though the normative concerns are familiar, there is more utility than simply appreciating how new technologies illuminate longstanding tensions in evidence law. This is because new technologies may also be proffered as potential options that could rebalance tradeoffs between values such as accuracy and due process.

### V.A. Weak Objections and Surmountable Challenges

#### V.A.1. That credibility assessment is exclusively a job for the jury

A plurality of the Supreme Court, led by Justice Thomas, stated in *Scheffer* that in ‘our criminal trial system … “the *jury* is the lie detector.” Determining the weight and credibility of witness testimony, therefore, has long been held to be the ‘part of every case [that] belongs to the jury, who are presumed to be fitted for it by their natural intelligence and their practical knowledge of men and the ways of men’.’^162^ Other courts have expressed similar sentiments.^163^ But why should evidence that ‘bears on’ credibility be so offensive to the system of trial by jury, particularly if juries are not very good at detecting when they are being lied to? (The analysis is slightly different for a ‘perfect’ memory detector, discussed *infra* Part V.C.) The modern role of the jury as a ‘lie detector’—rather than a broader conception of credibility assessor—was cemented in the seminal article by George Fisher.^164^ But Fisher’s focus was on the historical development of the jury as an ‘error-erasing’, legitimacy-preserving function.^165^ If the jury is not actually well-suited for the task of accurately assessing witness credibility—and if they already regularly consider other types of evidence that ‘bears on’ a witness or defendant’s credibility, such as character evidence^166^—a legal system prioritizing accurate results must seriously consider that even a ‘perfect’ credibility detector trumps ‘error-erasing’ concerns.

John Meixner argues that even if the role of credibility assessment is one exclusively for the jury, perhaps it should not be.^167^ Arguing that ‘this determination is inevitably based on the accuracy of jurors’ credibility determinations’, he concludes that because social science indicates that people (both trained experts and lay individuals) are not very good at detecting when another is lying to them, expert testimony on ‘the credibility of witnesses’ should be permitted toward the goal of fact-finding accuracy.^168^

The tension between the goals of accuracy and ‘error-erasing’ legitimacy (due to the perception of accuracy and the opacity of jury deliberations) is probably not solved by the type of memory detection device under consideration here—one that may still be vulnerable to inherent flaws in human memory. But the ‘credibility assessment is solely a task for the jury’ argument, without more, is weakest in that it ignores the fact that perceptions of accuracy feed directly into perceptions of legitimacy. If a perfect, or near-perfect, memory detection device was available, it is unlikely the public would perceive its exclusion in favor of assessment by a jury of peers as improving the accuracy of a verdict.

#### V.A.2. That the experts’ methods may have flaws or weaknesses

Brain-based memory detection results might be incorrect or misleading, because of human causes of ‘falsehood by design’.^169^ In the hands of the wrong person, perhaps motivated to find incriminating information in the brain of a given suspect, a brain-based assessment could be designed ‘in a way they know, or suspect, will lead a machine to report inaccurate or misleading information’—perhaps by choosing incriminating stimuli of which an innocent suspect is already aware, or by failing to include appropriate comparator stimuli. Even in the hands of an honest and well-meaning expert interested only in accurate results, stimulus design choice could be influenced—maliciously or inadvertently—by incomplete or inaccurate information about an investigator’s understanding of the events of interest. At present, all memory detection technologies start from a hypothesis about the ground truth, and assess whether that truth is recognized or recollected in the brain of a subject being imaged. Where that truth is unknown, or uncertain, or ambiguous—as in real-world settings—potential for human error in test design is magnified accordingly.^170^

Researchers also make choices about the degree of tolerance for uncertainty, risking a non-match to the one assumed by the fact finder, unless disclosed.^171^ In the memory detection studies reviewed in Part II, researchers made choices about the tolerances for false positives and false negatives in setting signal detection thresholds.^172^ Even if the tolerance for particular types of uncertainty is articulated to a fact finder, it is difficult to translate statistical thresholds in signal detection theory to concepts matching layperson judgments of certainty.^173^

As for an expert’s chosen analytic methods, consensus in functional brain imaging communities tends to exist in narrow groups. Different laboratory groups use varying degrees of proprietary code to run their experiments and analyze the data. Even in the broader brain imaging community, data collection and analytic techniques are far from standardized. A recent episode illustrates this plainly. Data analysis in the fMRI community recently received scrutiny in the popular press after an article comparing two different types of analyses stated that their findings ‘question the validity of some 40,000 fMRI studies and may have a large impact on the interpretation of neuroimaging results’.^174^ Though initially overstated and later corrected, the episode highlights that ‘the validity of fMRI data analysis paradigms has not been uniformly established and needs continued in-depth investigation. fMRI is a complex process that involves biophysics, neuroanatomy, neurophysiology, and statistics (experimental design, statistical modeling, and data analysis)…. Linking statistical methodology development and fundamental fMRI research is crucial for developing more accurate analysis methods, attributing accurate scientific interpretations to results, and ensuring the reliability and reproducibility of fMRI studies’. ^175^ When assessing reliability of methods—a necessary but not sufficient step toward credibility assessment—brain imaging software engineers and statisticians should be consulted as part of the relevant scientific community in determining the reliability not only of the behavioral and analytic method of data collection, but of the software implementing the collection and analysis methods.^176^ Although this consultation should be part of the admissibility analysis, credibility assessment requires that it is also addressed in front of the jury.

None of these problems are unique to brain-based memory detection evidence. Juries are presented with complicated scientific evidence all the time. Each of these problems may be addressable through ‘testimonial safeguards’, some of which are provided by rigorous peer review of the general methods and protocols that an expert relies upon, and others of which can be addressed by well-prepared cross-examination and opposing expert evidence.

#### V.A.3. That juries cannot be trusted to evaluate this complex scientific evidence about credibility

A final, surmountable objection to consider is actually an empirical claim. To this point, the argument against admissibility has been premised on an assumption that a sufficiently ‘reliable’ memory detection device would need to be nearly perfect in order to be sufficiently relevant and reliable. But what if the technique simply improves a fact finder’s guesses by providing some ‘useful’ or ‘helpful’ information, subject of course to cross-examination and opposing testimony exposing all the technology’s weaknesses? This analysis would cut in favor of its admissibility.

Two concerns arise: first, that a jury would overweight the memory detection evidence and it would be unduly prejudicial^177^; second, that they might wholly abdicate wrestling with the deliberative, explanatory process going on inside the jury room because an unexplainable machine assessed the credibility of a witness for them. But this is a testable hypothesis, and current evidence for the proposition that neuroscience-as-evidence is unduly persuasive to lay decision-makers is mixed at best.^178^ (Indeed, jurors may tend to ignore, rather than overweight, complex evidence that they do not understand, particularly when they feel they have their own intuitive assessment of witness credibility, as they do in their everyday lives.) Testing this hypothesis is difficult, but not impossible.^179^ In light of the incompleteness of the empirical data, there is little to definitively say other than that reasonable courts and scholars presently differ as to their intuitive judgments about whether jurors would be able to properly assess probabilistic evidence coming from impressive and relatively opaque technology accessing the mysterious inner workings of the human brain.

### V.B. Stronger Objections

#### V.B.1. That high reliability in memory detection may be biologically implausible

Here, we essentially apply the import of the scientific findings above. The current scientific consensus is that memory is inherently reconstructive, flexible, and malleable and that there is no form of storage that is a permanent ‘engram’ (memory trace) like a video recording or digital computer file. We now understand that memories undergo a process known as reconsolidation every time they are retrieved. That is, every time a stored memory trace is accessed and ‘reactivated’, it temporarily becomes ‘labile’, and hence prone to some degree of updating.^180^ While in this labile state, memory traces can be disrupted entirely by the administration of protein synthesis inhibitors or electroconvulsive shock therapy, or they can be modified by the introduction of new information.^181^ This is, in fact, the normal mechanism of learning. But it also means that memory detection cannot be thought of as accessing a file stored somewhere without recognizing that that file has potentially been modified by virtue of being accessed, every single time it is accessed.

Moreover, the most sophisticated brain-based memory detection techniques reviewed above confirm that false but subjectively believed memories may be so biologically similar to ‘true’ (veridical) memories that accuracy in fact-finding is truly limited. Furthermore, countermeasures and false positives may be unavoidable and undetectable. At present, many more types of countermeasures have not yet been investigated, such as the potential for deliberate false memory creation, forgetting via deliberate rumination on an alternate narrative, or implanted false memory creation via questioning or providing information. Finally, it is important to remember that here (as is true generally), the absence of evidence is probably not going to be evidence of absence. That is, the absence of a memory in a detection paradigm cannot confidently be said to represent a true absence of memory or actual experience. False negatives may be acceptable in certain diagnostic scenarios, but their presence as a boundary condition on the technology’s utility as forensic evidence should be appreciated.

#### V.B.2. That even highly reliable memory detection may be so technologically complex as to be impenetrable to machine credibility assessment

The technological and biological complexity of sophisticated brain-based memory detection makes it exceedingly difficult—perhaps impossible—for mere laypersons to assess whether they should ultimately believe it as fact. This brings us to the second layer of credibility analysis for memory detection evidence. Even if a memory detection device, or a lie detection device, could perform reliably enough—that is, it satisfying repeated validation studies—such that it was normatively preferable (and assuming for the moment that accuracy is the only valued metric) to a jury in terms of assessing witness credibility, how do we know we should believe what the device says, or how much weight to give to the result, or the expert opinion based on it? Because the ultimate question upon which the test purports to aid the jury is one the jury is squarely charged with, the jury must also be able to assess the credibility of the machine and test itself.

‘Machine credibility’, a concept elucidated by Andrea Roth, is whether the fact finder draws the correct inference from information conveyed by a machine source; in short, ‘the machine’s worthiness of being believed’. ^182^ Courts and most scholars have not yet recognized that machines themselves can provide evidence that ‘merits treatment as credibility-dependent conveyances of information’.^183^ Just as credibility testing of human witnesses encompasses more than simply assessing sincerity or mendacity—of which machines are theoretically incapable—Roth argues that ‘the coherence of ‘machine credibility’ as a legal construct depends on whether the construct promotes decisional accuracy’.^184^ Limiting for the moment the task of credibility assessment to one of decisional accuracy, what this translates to in practical terms is how much ‘weight’ a jury should give to a machine conveyance, just as a jury is entitled to give varying weight to witness testimony.

Brain-based memory detection techniques fall squarely within the realm of credibility-dependent machine conveyances. They essentially convert the biological contents of a human skull (and, by extension, the subjective contents of a human mind) to an assertion by a machine, reported by a human expert. In the case of machine learning applied to fMRI or EEG data, the assertion is that the contents of a person’s mind, in response to certain stimuli presented, are a pattern match, within a given degree of confidence, to a subjective experience of recognition or recollection.^185^ Such output merits treatment as a credibility-dependent conveyance of information, even though it is ultimately presented to the jury by an expert. ‘Just as human sources potentially suffer the so-called ‘hearsay dangers’ of insincerity, ambiguity, memory loss, and misperception’, machine-learning algorithms interpreting raw brain data potentially suffer ‘black box’ dangers that could lead a fact finder to draw the wrong inference from information conveyed by a machine source…. Just as the ‘hearsay dangers’ are more likely to arise and remain undetected when the human source is not subject to the oath, physical confrontation, and cross-examination, black box dangers are more likely to arise and remain undetected when a machine utterance is the output of an ‘inscrutable black box’.^186^

Recall that fMRI-based lie detection methods are using machine learning in the form of MVPA to extract more and better information from subtle patterns in brain imaging data.^187^ It is the technological sophistication of these methods that are revealing the biological limits of memory detection. But the technology itself brings evidentiary risks that should be the basis of strong objections to courtroom use.

Andrea Roth’s taxonomy of machine credibility warns of falsehood by ‘machine learned design’.^188^ In the most advanced forms of brain-based memory detection, testimonial safeguards in the form of front-end protocol design may not be feasible, as ‘[d]ata scientists have developed very different ‘evaluation metrics’ to test the performance of machine-learning models depending on the potential problem being addressed’.^189^ Human selection goes into choosing machine-learning algorithms and some of their parameters for training, but the algorithms themselves may present a form of opacity because of the ‘mismatch between mathematical procedures of machine-learning algorithms and human styles of semantic interpretation’^190^—that is, the mechanisms of machine learning may not map neatly onto humans’ ability to explain them.

Machine learning is a critical part of the most advanced research into brain-based memory detection. Rissman and colleagues, in the first paper to apply MVPA to fMRI-based memory detection analysis, explored several machine-learning algorithms, ‘including two-layer back-propagation neural networks, linear support vector machines, and regularized logistic regression [RLR]’, electing the latter after they ‘found that RLR generally outperformed the other techniques, if by only a small amount’ in terms of classification accuracy.^191^ This is, of course, a human design element—choosing which type of machine-learning algorithm to use.^192^ Other types of classifiers are available and used by fMRI researchers interested in the detection of memory and intention. Peth and colleagues chose a linear support vector machine,^193^ and a 2014 fMRI study from yet another research group attempting to decode true thoughts independent of an intention to conceal used a Gaussian Naïve Bayesian classifier.^194^ Each type of classifier must first be ‘trained’ on data fed to it by a human—another source of potential inferential error, given the number of assumptions about shared features of training data and test data of interest—the result of which is a ‘matrix of weights that will then be used by the classifier to determine the classification for new input data’.^195^ The classifier itself can report its degree of certainty in its classification decision, if given no particular threshold by its human programmers.^196^

The choice of machine-learning algorithm by a particular set of researchers or forensic designers could have an impact on its interpretability. Although ‘machine learning models that prove useful (specifically, in terms of the “accuracy” of the classification) possess a degree of unavoidable complexity’, different machine-learning algorithms have different levels of opacity.^197^ That is, different algorithms may have different degrees of ‘black-boxiness’ in the sense that they may be able to be subjected to credibility testing. In a linear classifier, such as RLR and linear support vector machines, it is possible to know roughly how much each brain voxel matters to the ultimate classification decision. Indeed, researchers using these classifiers can create ‘importance maps’ of voxels, which themselves could be sort of a window into the machine’s ‘thinking’.^198^ A neural network classifier with multiple hidden layers would be less ‘knowable’ in terms of how the machine is thinking, because the knowledge relevant to the classification decision is both distributed and non-linear.^199^ That is, some classifiers may be more ‘interpretable’ than others, and suggests a ‘testimonial safeguard’ for brain-based memory detection technologies that use machine-learning classifiers that reveals, in terms or diagrams laypeople can understand, how the classifier made the decision.^200^

It is an empirical question whether such logic could be sufficiently explained to laypersons via direct or cross-examination (including whether the expert witnesses themselves even fully understand the machine’s classification decisions), or whether certain front-end procedures and protocols, including model jury instructions, could be designed to prepare and prime a jury to engage in sufficient scrutiny of a machine-learning-based expert conclusion.^201^ Pretrial disclosure and access to raw data, analysis code, and even the scanner itself could potentially expose flaws leading to inaccurate inferences,^202^ but such ‘adversarial design’ also assumes an expensive degree of expertise available to both sides.

But this empirical question is even more difficult to investigate than the objection above that jurors might not understand complex science—especially where it may be impossible for an expert to describe how the algorithm works, other than that it does, and therefore it should be trusted. Does this matter, in the sense that validation studies showing that ‘it works’ should be good enough for technological black boxes of any kind? That may be generally true and is certainly asserted by some proponents of technology in law,^203^ but recall that sufficient validation studies in the memory detection context might be impossible in situations where the ground truth is truly disputed and cannot be known or otherwise corroborated. This is not a type of expert evidence where trial court acceptance of ‘tacit expertise’ should be permitted to suffice for validation studies.^204^ Moreover, meaningful validation studies of error rates and diagnostic value will depend critically on base rates of false/inaccurate memories in analogous situations, which are not currently and may never be able to be known.^205^

### V.C. Witness Personhood and Evidentiary Relativism: Philosophical and Normative Considerations

#### V.C.1. Philosophical

If we had a ‘perfect’ brain-based memory detector, should we use it? There are values besides decisional accuracy that the system of trial by jury with testimonial evidence embodies.^206^ While the plurality of values may be familiar,^207^ are they uniquely implicated by jury assessment of memory detection evidence?

Our answer aligns with the intuition first articulated by Justice Linde of the Oregon Supreme Court: ‘I doubt that the uneasiness about electrical lie detectors would disappear even if they were refined to place their accuracy beyond question. Indeed, I would not be surprised if such a development would only heighten the sense of unease and the search for plausible legal objections’.^208^ Having made the case above that memory detection is, for intents and purposes of credibility assessment, the same as lie detection, this section attempts to probe that intuition that in part motivated this article: that more is at stake in directly assessing witness memories than in most other forms of expert evidence, and these values may not be addressable by testimonial safeguards, adversarial design, or other tweaks for assessing machine credibility.^209^ The strongest direct critique of Justice Linde’s argument is that accurate fact determination should be the dominant value in assessing evidence that can go to the jury, superseding all other considerations in the event that polygraphs (and, by extension, memory detection) became perfect.^210^ But this is not a strong enough rebuttal to entirely set the personhood issue aside (discussed below), particularly in light of the fact that the scientific findings may never be able to reveal perfect truth because of the reconstructive nature of memory.

Perhaps it is the lack of explainability that is what is intuitively troublesome here—that we do not really know how memory works, and we may not be able to explain how memory detection works, but we might be willing to rely on it to assess the credibility of witnesses. We have claimed that memory detection devices may be impenetrable to credibility assessment if they are truly black boxes involving certain types of machine learning that cannot be adequately explained. What is lost, with respect to the adversarial trial context, if brain-based memory detection is (at some level) unexplainable?

Here we can look to the expanding literature considering automation in other aspects of legal decision-making. Kiel Brennan-Marquez recently argued in the context of probable cause that plausibility—the ability to ‘explain’ a probable cause decision—has independent value as a check on state power.^211^ Yet juries are not only not required to explain their verdicts, but any explanations cannot be used as a basis for appeal, save for a few narrow circumstances.^212^ Juries themselves are to act as a ‘black box’, for the sake of finality in conflict resolution, and only the inputs are constrained by the rules of evidence. Yet there is explanation going on, one assumes, inside the jury room during the deliberative process. It is perhaps this value, which Brennan-Marquez identifies as ‘prudence’, that we seek to preserve by avoiding jury abdication of credibility assessment by acceptance of a machine and expert’s say-so.

Is there more than the preservation of prudence within the jury deliberation room? Andrew Selbst and Solon Barocas recently identified three different values in opening the black box of machine-learning algorithms involved in legally relevant decision-making (though not as trial evidence *per se*). The first is ‘a fundamental question of autonomy, dignity, and personhood’—explanation as an inherent good.^213^ The second is instrumental: explanation as enabling future action of those that are subject to machine decisions. The third is about justification and exposing a basis for *post hoc* evaluation. Of these, only the first is relevant to our assessment of juror reliance on machine-based expert testimony in their decision-making, which is too many steps removed to inform the future action of witnesses and defendants, and which cannot be exposed to justification and *post hoc* evaluation, save for a few narrow reasons.^214^ Selbst and Barocas link the personhood rationale to the concept of procedural justice, which Tom Tyler has demonstrated is a necessary condition for legitimacy in the legal system.^215^ But is the personhood—the fallible, imperfect, reconstructed memories that constitute the person and underpin their ability to narrate their own story—of a ‘witness’ evaluated by a jury really so key to perceptions of procedural legitimacy?

Our tentative answer is yes. It seems that what may be at stake may be the fundamental value of personhood—as opposed to the reductionist, objectified readout of one’s brain—and its function as a cornerstone of procedural justice.^216^ This takes seriously the personhood not only of witnesses, but also of jurors, and their ability to appreciate the personhood of a witness whose credibility they must assess.^217^ The version of the personhood argument we adopt is specially informed by the findings of memory detection research and memory research more generally: that recollection of autobiographical experience that defines our personhood is not machine-like at all, but rather imperfect, dynamic, and reconstructive. Our autobiographical memories, and our subjective recollection and constant re-interpretation of them, is a fundamental part of our identity. Indeed, those who lose their memories can also lose their very sense of a dignified self.^218^ Of course, this answer may not justify a blanket prohibition on memory detection evidence on the grounds of ‘personhood’, because the risks depend upon who is offering the evidence (eg a defendant volunteering exculpatory evidence, whose personhood is not threatened by exercising their autonomy) and the legal issues and theory of the case, as discussed above.

Personhood is in fact central to witness credibility, as evidenced by the history of witness competency rules and existing doctrine of impeachment.^219^ The dark side of this is that social and behavioral status has long been—and still is in character-based impeachment doctrine^220^—a proxy for who is worthy of belief, with troubling disproportionate effects on persons of color and communities without privilege.^221^ Julia Simon-Kerr suggests that this state of affairs means that, if reliable, lie detection science—presumably a more objective way of establishing truth—should replace status-based assessments of credibility in terms of impeachment doctrine.^222^ But if the best we can do with science-based techniques is identify the biological correlates of a ‘subjective’ experience—filtered through the impressions and decisions of whomever is probing the witness’s brain—are we really any closer to establishing objective truth, or are we now assessing witness credibility based on the social status of the expert and her sophisticated machine? This situation may in fact be normatively preferable to discriminatory proxies for credibility that rely on racist, sexist, and classist devaluation of persons, but it is arguably no closer to respecting the dignity and personhood of the witness as the narrator of their own memory and experience.

Putting these pieces together: if personhood (of some sort, derived from sincere narration of subjective experience of memory) is critical to witness credibility, and assessments of personhood/credibility are critical to the procedural legitimacy of juries, and this legitimacy comes from a belief (a myth?) that juries engage in a prudent and deliberative, though opaque, process of credibility assessment, then brain-based memory detection directly challenges this edifice of trial by jury. Again, Justice Linde intuited this conclusion:

One of these implicit values surely is to see that parties and the witnesses are treated as persons to be believed or disbelieved by their peers rather than as electrochemical systems to be certified as truthful or mendacious by a machine…. A machine improved to detect uncertainty, faulty memory, or an overactive imagination would leave little need for live testimony and cross-examination before a human tribunal; an affidavit with the certificate of a polygraph operator attached would suffice. There would be no point to solemn oaths under threat of punishment for perjury if belief is placed not in the witness but in the machine.^223^

Justice Linde and other commentators go so far as to suggest that a perfect lie detector—and by extension, a memory detection device—would render the jury entirely superfluous.^224^ This ‘witness personhood’ concept suggests that to the extent we take the jury system seriously, we should resist introducing even ‘perfect’ black box brain-based memory detection evidence into the jury black box.

#### V.C.2. Normative

Evidentiary value is not a static quality in terms of probativeness, relevance, or reliability; it is inherently a relative quality. Normative assessment of the utility and permissibility of a particular piece of evidence must be context-driven. Sometimes, that context is one of alternatives much more horrific than premature or even ‘junk’ science.

Recall from the start of this article that about a decade ago, the international press reported on a murder story from India. Aditi Sharma had been accused of murdering her fiancé, Udit Bharati, with poison given to him in sweets. She was convicted largely on the basis of evidence from a brain-based memory detection system (here, the BEOS) that demonstrated her ‘experiential knowledge’ in response to statements read by investigators implicating her in the crime. According to initial news reports, the BEOS technology was neither published nor peer reviewed, but its inventors ‘claim[ed] the system can distinguish between peoples’ memories of events they witnessed and between deeds they committed’.^225^ At the time, two Indian states had set up labs where BEOS could be used by investigators, and one in Maharashtra reported that over 75 suspects and witnesses had undergone the test in less than 2 years.^226^ Of those, at least 10 resulted in confessions.^227^

From what we know of the test, BEOS uses a proprietary set of 11 electrical signals in the brain from 30 EEG probes on the surface of the scalp to make up the ‘signature’ of experiential, first-hand, participatory ‘remembrance’ of an event, as differentiated from ‘knowledge’ or ‘recognition’ gained by learning about an event from another source.^228^ Dr Champadi Raman Mukundan, a former university scientist, developed BEOS in conjunction with Axxonet, a private software-as-a-service company, partially at the request of Indian law enforcement authorities and India’s National Institute of Mental Health and Neuro-Sciences, and partially out of Dr Mukundan’s own interest in and frustration with the EEG-based P300-related techniques.^229^

The technologies were not deployed without scientific skepticism in India. A month before Sharma’s conviction, a six-member peer review committee headed by India’s National Institute of Mental Health and Neuro-Sciences concluded that both BEOS and what they called ‘brain mapping’ (which refers to the ‘Brain Fingerprinting’ EEG-based technology promoted by Lawrence Farwell)^230^ were ‘unscientific and … recommended against their use as evidence in court or as an investigative tool’.^231^ This report was delivered to India’s chief forensic scientist, Dr MS Rao, who critiqued it for the committee’s failure to visit the forensic laboratories using BEOS. Dr Rao claimed that the technique’s results were ‘encouraging’, based upon results in actual cases.^232^ Upon learning of the legal application of the BEOS technology in the Sharma case, US-based commentators critiqued the ‘shaky’ science for lack of peer review and ‘variously called India’s application of the technology to legal cases “fascinating,” “ridiculous,” “chilling,” and “unconscionable”’.^233^

The current status of forensic brain-based memory detection in India is mixed. In May 2010, the Indian Supreme Court held that results of ‘deception detection tests’ including the Brain Electrical Activation Profile test^234^ are not admissible, and neither are evidentiary fruits of such tests administered in an investigative context but without a subject’s voluntary consent.^235^ Nevertheless, recent media and articles suggest that brain-based tests, including BEOS, are still being used in investigation and prosecution in lower courts, possibly as a result of coerced consent.^236^ Even after the 2010 Supreme Court judgment, Indian forensic labs planned to continue their expansion of brain scanning techniques.^237^

Why do brain-based investigation techniques persist in the absence of formal evidentiary admissibility, and the presence of significant scientific skepticism? The context of Indian policing is the key to the answer: the status quo alternative is too often literal torture. Indian policing is ‘rife with stories of physical torture and custodial deaths’.^238^ BEOS proponents have suggested that even scientifically doubtful interrogation techniques may be a lesser of two evils.^239^ Indeed, a BEOS technician at the Directorate of Forensic Science in Mumbai, said in an interview with Wired that subjects—even those accused of murder—are ‘so, so relieved to be here. They’re so happy to be here with us, because we’re not scary. We talk to them nicely. Just imagine… you can imagine in India the way the police must be dealing with them’.^240^ Jinee Lokaneeta argues that in the context of brutal Indian criminal investigations and a disconnect between the law articulated by courts and routine impunity for police violence, courts have determined that the necessity of scientific evidence trumps concerns about scientific reliability.^241^

This context takes the discussion of investigatory use of brain-based memory detection completely beyond the realm of scientific reliability and toward moral relativism. In a situation where the outcome would be the same—conviction and confinement—less intrusive means are obviously preferable. That is, an inaccurate brain-based test leading to a confession is morally preferable to torture leading to the same confession, even if that confession is factually wrong in both cases. In the aggregate, it is an empirical matter; it is not possible to say with any degree of certainty that inaccurate scientific methods of investigation would lead to ‘more’ wrongful convictions than a regime of torture-based interrogation.

Nevertheless, this is not to advocate for routine investigatory or courtroom use in the USA. Indeed, in a system where the vast majority of criminal charges are resolved by plea bargain—with many of those based on inadmissible evidence, as discussed below—there is not an obvious ‘greater evil’ of torture as the status quo to justify deployment of memory detection technology in investigations and, ultimately, plea bargaining. This calculus may be different in situations of urgent national security concerns or other truly exigent circumstances, but scientific limitations may exist there that make the utility of such application doubtful.^242^

## VI. MEMORY DETECTION IN FREE-PROOF SYSTEMS: INVESTIGATIONS AND ADMINISTRATIVE ADJUDICATIONS

Finally, how should brain-based memory detection be evaluated in free-proof systems, unconstrained by evidentiary rules because no jury is present? Application to investigative or administrative proceedings may be both legally permissible and normatively preferable, given the relaxed procedural requirements and increased specialization of decision-makers using the evidence. These less-formal contexts liberate experts from having to provide perhaps uncollectible data about real-world error rates, and from obtaining ‘general acceptance’ of the use for forensic purposes. Residual uncertainties about technological accuracy may not weigh so heavily, especially where the technology is presented as the only corroboration to thin testimony, or an alternative to methods known to be inaccurate, harmful, or abusive. Moreover, the decision-makers evaluating proffered evidence in investigations or administrative proceedings are not juries to be shielded from unreliable or un-assessable evidence, but experienced practitioners, who are often assumed—but not conclusively shown—to be less susceptible to reliance on inadmissible evidence.^243^ Should we worry about brain-based memory detection used in a free-proof system, but with experienced decision-makers? Or should we hope for it?

Our answer is a qualified, and perhaps unsatisfying: maybe. Although it may be the case that investigatory and administrative settings lack the adversarial elements contributing to the ‘central myth’ of trial as a determination of truth,^244^ and more contextual flexibility is available, the accuracy and limitations of memory detection technology must still be well-characterized to justify deployment in investigatory or administrative contexts. Even though the legal hurdles (or guardrails) are gone, the limitations of memory detection derived from the biological boundary conditions on accurate, veridical memories remain.

Investigative use of memory detection technology is not only easy to foresee, it is being actively pursued^245^ and, in some jurisdictions, already in practice. In the USA, inadmissible technologies are routinely used in investigations.^246^ Internationally, brain-based (and behavior-based) memory detection technology is currently used in a handful of jurisdictions.^247^ Investigatory use of memory detection, untested by evidentiary hearings and cross-examination, may be an avenue to unwarranted plea deals or inaccurate confessions.^248^ Moreover, the specter of false confessions is an unexplored area in memory detection research. Are subjectively experienced memories of an event created during the type of interrogations that result in false confessions, where investigators may supply details and suggestions? Based on current understanding of memory, it is definitely plausible and perhaps even likely. It is at this point impossible to know whether such an implanted memory would be detectable for what it is, or biologically indistinguishable from a true memory for the event. In such a scenario, the memory detection technology risks becoming a set of shackles rather than a neutral tool for fact development.

Application to administrative proceedings where credibility is central is also possible to imagine, such as asylum hearings and civil commitment proceedings. As to the former, an application for asylum can hinge upon credibility determinations made by trained officers and judges.^249^ Credibility determinations often founder on an applicant’s inconsistencies in the retellings of their experiences, notwithstanding research indicating that discrepancies in autobiographical accounts are common among ordinary people, and more common among those who have suffered trauma.^250^ If a memory detection expert could design a set of stimuli to detect whether an applicant had truly experienced events amounting to persecution, brain-based memory detection could potentially be a superior and consistent way to adjudicate such claims, free from the human biases and inconsistences between asylum officers. But claims that machines are less biased than humans should be viewed with suspicion, mindful of the rapidly expanding literature on algorithmic bias in many areas of automated decision-making.^251^ Brain-based memory detection using machine learning has not even begun to be assessed for such forms of bias, nor is it immediately obvious what kind of human-like biases might infect algorithmic brain-based memory detection.

## VII. CONCLUSION

This article has reviewed the current science of memory detection and ultimately argued that courtroom admissibility is a misdirected pursuit of that technology. At present, its admissibility would be precluded under *Daubert*, *Frye*, or state equivalents, primarily for lack of known error rates and lack of ‘general acceptance’ in the relevant scientific communities. But we have further argued that even if such error rates and acceptance accumulated, the most sophisticated brain-based memory detection devices may still not be suitable for courtroom use. This is first because the most advanced brain-based memory detection work suggests that only subjective experiences, rather than objective truths, may be accessible, rendering memory detection generally on the same footing as sincerity detection. Second, the method of acquiring that information requires machine-learning algorithms that may be opaque or even unexplainable to a jury, hindering their ability to assess the machine (and expert’s) credibility and assign appropriate weight. And even if a memory detection device worked perfectly well, would we want to use it? We have argued that if we take the jury system seriously, brain-based memory detection as evidence risks eliding the personhood of witnesses and thus undermining systemic, procedurally based legitimacy.

It is fair to ask whether we are letting the perfect be the enemy of the good-enough, or whether closing (but perhaps not barring) the doors to using brain-based memory detection as courtroom evidence will stagnate the interdisciplinary research enterprise. As to the latter, this concern is misplaced. There is still much to discover about the workings of memory itself, and prudent researchers are using the most sophisticated techniques to understand its contours and boundaries, rather than being driven by forensic application. As to the former, we think that prudence in fact-finding application is nevertheless warranted, and that systemic application of the knowledge of human memory gained from such work would have an overall greater effect than the handful of cases per year that would be litigated involving brain-based memory detection. That is, the advancing knowledge about human memory that is accumulating from memory detection research may be an aid to future evidence rulemaking, if not to individual fact-finding, just as research on eyewitness memory has finally begun to influence courtroom procedures and jury instructions.^252^

## Footnotes

1 State of Maharashtra v. Sharma (June 12, 2008), Case No. 508/07, Sessions Court, Pune (India) (copy of decision on file with ERDM).

2 Anand Giridharadas*, India’s Novel Use of Brain Scans in Courts is Debated*, N.Y. Times, Sept. 14, 2008, at A10; see also Nitasha Natu, *This Brain Test Maps the Truth*, Times India, https://timesofindia.indiatimes.com/city/mumbai/This-brain-test-maps-the-truth/articleshow/3257032.cms (accessed Mar. 3, 2020) (reporting on the Sharma case and another murder case in which the accused was convicted of a brutal murder and robbery after a ‘BEOS test [was] positive,’ though a lead prosecutor stated that ‘while BEOS was a useful technique of examination, it could not achieve conviction by itself. “The technique needs to be corroborated with other evidence”’.)

3 Angela Saini, *The Brain Police: Judging Murder with an MRI*, Wired (May 27, 2009), https://www.wired.co.uk/article/guilty.

4
*Id.*

5 See eg Jennifer S. Bard, *“Ah Yes, I Remember It Well”: Why the Inherent Unreliability of Human Memory Makes Brain Imaging Technology A Poor Measure of Truth-Telling in the Courtroom*, 94 Or. L. Rev. 295, 351 (2016); Teneille Brown & Emily Murphy, *Through a Scanner Darkly: Functional Neuroimaging as Evidence of a Criminal Defendant’s Past Mental States*, 62 Stan. L. Rev. 1119 (2010); Brian Farrell, *Cannot Get You Out of My Head: The Human Rights Implications of Using Brain Scans as Criminal Evidence*, 4 Interdisc. J. Hum. Rts. L. 101 (2010); Lyn M. Gaudet & Gary E. Marchant, *Under the Radar: Neuroimaging Evidence in the Criminal Courtroom*, 64 Drake L. Rev. 577 (2016); J.R.H. Law, *Cherry-Picking Memories: Why Neuroimaging-Based Lie Detection Requires A New Framework for the Admissibility of Scientific Evidence Under FRE 702 and Daubert*, 14 Yale J.L. & Tech. 1 (2011); Michael L. Perlin, *“His Brain Has Been Mismanaged with Great Skill”: How Will Jurors Respond to Neuroimaging Testimony in Insanity Defense Cases?*, 42 Akron L. Rev. 885 (2009); Mark Pettit, Jr., *FMRI and BF Meet FRE: Brain Imaging and the Federal Rules of Evidence*, 33 Am. J.L. & Med. 319 (2007); Francis X. Shen et al., *The Limited Effect of Electroencephalography Memory Recognition Evidence on Assessments of Defendant Credibility*, 4 J.L. & Biosciences 330, 331 (2017); William A. Woodruff, *Evidence of Lies and Rules of Evidence: The Admissibility of FMRI-Based Expert Opinion of Witness Truthfulness*, 16 N.C. J.L. & Tech. 105 (2014); So Yeon Choe, *Misdiagnosing the Impact of Neuroimages in the Courtroom*, 61 UCLA L. Rev. 1502 (2014); Eric K. Gerard, *Waiting in the Wings? The Admissibility of Neuroimagery for Lie Detection*, 27 Dev. Mental Health L. 1 (2008); Jennifer Kulynych, *Psychiatric Neuroimaging Evidence: A High-Tech Crystal Ball?*, 49 Stan. L. Rev. 1249 (1997); Christina T. Liu, *Scanning the Evidence: The Evidentiary Admissibility of Expert Witness Testimony on MRI Brain Scans in Civil Cases in the Post-Daubert Era*, 70 N.Y.U. Ann. Surv. Am. L. 479 (2015); Adam Teitcher, *Weaving Functional Brain Imaging into the Tapestry of Evidence: A Case for Functional Neuroimaging in Federal Criminal Courts*, 80 Fordham L. Rev. 355 (2011). For further work on the impact and persuasiveness of brain imaging technology, see note 175.

6 See John B. Meixner, Jr., *Admissibility and Constitutional Issues of the Concealed Information Test in American Courts: An Update*, *in* Detecting Concealed Information and Deception 405, 406 (J. Peter Rosenfeld ed., 2018) (arguing that the evidentiary and constitutional limitations on the courtroom admissibility of versions of the Concealed Information test ‘should drive the research agenda of every CIT researcher interested in the practical use of their work.’); Gerson Ben-Shakar & Mordechai Kremnitzer, *The CIT in the Courtroom: Legal Aspects*, *in* Memory Detection: Theory and Application of the Concealed Information Test 276 (Bruno Verschuere et al. eds., 2011) (arguing that the CIT has the potential of meeting *Daubert* criteria for admissibility); J. Peter Rosenfeld et al., *Detection of Concealed Stored Memories with Psychophysiological and Neuroimaging Methods, in* Memory and Law 264, 290 (Lynn Nadel & Walter Sinnott-Armstrong eds., 2012) (providing recommendations that ‘would lead to enhanced implementation of the CIT and possibly also to its use as admissible evidence in courts’).

7 Interview with CR Mukundan, Inventor and Patent of the BEOS Profiling Technology, in Bangalore, India (Aug. 11, 2009); see also John Meixner, *Liar Liar: Jury’s the Trier? The Future of Neuroscience-Based Credibility Assessment in the Court.* 106 Nw. U. L. Rev. 1451, 1474–75 (2012).

8 Reviewing P300 complex trial protocol techniques in 2013, Rosenfeld et al. wrote that once the P300 CIT procedure was validated in a ‘field population,’ it would satisfy all *Daubert* criteria for admissibility. J. Peter Rosenfeld et al., *Review of Recent Studies and Issues Regarding the P300-Based Complex Trial Protocol for Detection of Concealed Information*, 90 Int’l J. Psychophysiology 118 (2013); see also Ben-Shakar & Kremnitzer, *supra* note 6.

9 See text accompanying notes 226–241, *infra.*

10 Memory matters to other actors in the legal system, including jurors and judges. See eg Anders Sandberg et al., *The Memory of Jurors: Enhancing Trial Performance*, *in* Memory and Law, *supra* note 6, at 213. But the categories of witnesses and suspects are most apt for evidentiary analysis, yet broad enough to encompass a range of witness types in both civil and criminal proceedings (such as victims, percipient witnesses, character witnesses, defendants, and co-conspirators), as well as inclinations (hostile, friendly, self-serving, or reluctant).

11 Jesse Rissman & Emily R.D. Murphy, *Brain-based Memory Detection and the New Science of Mind Reading,* in The Oxford Handbook of Human Memory (Michael J. Kahana & Anthony D. Wagner eds.) (forthcoming 2020).

12 See *infra* note 146 and accompanying text.

13 In 2011, the New Jersey Supreme Court substantially modified the state’s procedures for evaluating eyewitness identifications after appointing a Special Master to evaluate the scientific evidence behind such identifications. State v. Henderson, 27 A.3d 872 (N.J. 2011). After *Henderson*, New Jersey defendants who showed some evidence of ‘suggestiveness’ in a prosecution’s eyewitness identification are entitled to a pretrial hearing to evaluate the variables affecting that identification and decide its admissibility. The ‘Henderson’ court also requested that committees draft proposed revisions to model jury instructions on eyewitness identification that would address the myriad variables affecting identifications in order to guide how a jury should weigh the identification if it is admitted, ultimately reducing reliance on expensive and time-consuming expert testimony to explain to jurors the scientific consensus on how human memory works and the factors that affect it. *Id.* at 878. The report and recommended jury instructions, ultimately adopted in 2012, are available at https://njcourts.gov/courts/assets/criminal/outofcourtreport.pdf. In 2019, the New Jersey Supreme Court modified the holding from ‘Henderson’ to entitle defendants to a pretrial hearing on the admissibility of a witness’ identification even without evidence of suggestiveness on the part of law enforcement, in circumstances where no electronic or contemporaneous, verbatim written recording of the identification procedure was prepared. State v. Anthony, 204 A.3d 229 (N.J. 2019). Other states have taken similar steps. In 2014, Pennsylvania’s Supreme Court held that expert testimony regarding eyewitness identification was no longer per se inadmissible, but rather a matter of trial court discretion. Commonwealth v. Walker, A.3d 766 (Pa. 2014). But efforts to implement model jury instructions similar to New Jersey’s (drafted in 2012) do not appear to have advanced further. See Jeannine Turgeon et al., *Crafting Model Jury Instructions for Evaluating Eyewitness Testimony,* The Pa. Law., Sept./Oct. 2014, at 49; *see* also State v. Guilbert, 306 Conn. 218, 234 (Conn. 2012) (disavowing earlier rulings which restricted expert testimony, stating that such previous rulings are ‘out of step with the widespread judicial recognition that eyewitness identifications are potentially unreliable in a variety of ways unknown to the average juror’.).

14 Daniel V. Meegan, *Neuroimaging Techniques for Memory Detection: Scientific, Ethical, and Legal Issues*, 8 Am. J. Bioethics 9 (2008) (commentaries follow the piece, on 21–36). In 2008, Daniel Meegan comprehensively reviewed neuroimaging techniques for memory detection, critically evaluating then-existing studies along four attributes essential to an ideal forensic memory detection test: specificity (the degree to which an effect in a test is specific to the stimuli of interest, like the murder weapon versus generic weapons in the same category), retrieval automaticity (the extent to which a memory retrieval is automatic, and thus presumed to be resistant to a countermeasure by attempting to respond to the stimulus as if it were new), encoding flexibility (the extent to which a memory detection test gives robust results in varied encoding conditions, closely related to the sin of ‘absent-mindedness’), and longevity (the length of time that an effect remains measurable after the original encoding of the memory, also known as the sin of ‘transience’).

15 See generally Henry T. Greely & Judy Illes, *Neuroscience-Based Lie Detection: The Urgent Need for Regulation*, Am. J.L. Med. 377 (2007); see also sources *infra* note 127; U.S. v. Semrau, 693 F.3d 510 (6th Cir. 2012).

16 A memory ‘trace’ is the physical record in the brain of a past experience. This is sometimes represented by the concept of the ‘engram’. *See* eg Lucas Kunz, et al., *Tracking Human Engrams Using Multivariate Analysis Techniques*, *in* 28 Handbook of Behavioral Neuroscience 481–508 (Denise Manahan-Vaughn, ed., 2018); Sheena A. Josselyn, Stefan Köhler, & Paul W. Frankland, *Finding the Engram*, 16 Nature Rev. Neurosci. 9, 521–34 (2015).

17 Meegan, *supra* note 14, at 9.

18 The CIT stands in contrast to a protocol called the Comparison Question test. *See* Rosenfeld et al., *supra* note 8.

19 Other research does attempt something closer to mind-reading. See eg Russell A. Poldrack, The New Mind Readers: What Neuroimaging Can and Cannot Reveal about Our Thoughts (2018); P.R. Roelfsema et al., *Mind Reading and Writing: The Future of Neurotechnology*, 22 Trends in Cognitive Sciences 598 (2018); T. Horikawa et al., *Neural Decoding of Visual Imagery During Sleep*, 340 Science 639, 639–42 (2013); U.S. Patent 9,451,883 (filed Dec. 21, 2012), https://patents.google.com/patent/US9451883B2/en; S. Nishimoto et al., *Reconstructing Visual Experiences from Brain Activity Evoked by Natural Movies*; 21 Current Biology 1641, 1641–66 (2011).

20 This is the working theory of the inventor of BEOS, see discussion accompanying notes 228–233, *infra.*

21 See Alan S. Brown, *A Review of the Déjà Vu Experience*, 129 Psychol. Bull. 394 (2003).

22 See eg Ira E. Hyman Jr. & Elizabeth F. Loftus, *Errors in Autobiographical Memory*, 18 Clinical Psychol. Rev. 933, 933–94 (1998); Elizabeth F. Loftus & Hunter G. Hoffman, *Misinformation and Memory: The Creation of New Memories*, 118 J. Exp. Psychol. 100 (1989).

23 Hung-Yu Chen et al., *Are There Multiple Kinds of Episodic Memory? An fMRI Investigation Comparing Autobiographical and Recognition Memory Tasks*, 37 J. Neurosci. 2764 (2017); see also Tiffany E. Chow et al., *Multi-voxel Pattern Classification Differentiates Personally Experienced Event Memories from Secondhand Event Knowledge*, 176 NeuroImage 110 (2018).

24 The 2003 National Research Council Report on the polygraph test, which traditionally uses autonomic nervous system measurements such as heart rate, blood pressure, and skin conductance, noted that ‘[c]ountermeasures pose a serious threat to the performance of polygraph testing because all the physiological indicators measured by the polygraph can be altered by conscious efforts through cognitive or physical means.’ Nat’l Research Council, The Polygraph and Lie Detection 3 (2003), https://www.nap.edu/read/10420/chapter/2#3. The putative inventor of the ‘guilty knowledge test’ suggested that brain waves would be less vulnerable to countermeasures, largely because of their temporal brevity: ‘Because such potentials are derived from brain signals that occur only a few hundred milliseconds after the [guilty knowledge test] alternatives are presented… it is unlikely that countermeasures could be used successfully to defeat a GTK derived from the recording of cerebral signals’. David T. Lyyken, A Tremor in the Blood 293 (1998).

25 See generally Daniel Schacter, The Seven Sins of Memory: How the Mind Forgets and Remembers (2002).

26 See generally Jamie DeCoster & Heather M. Claypool, *A Meta-Analysis of Priming Effects on Impression Formation Supporting General Model of Informational Biases*, 8 Personality Soc. Psychol. Rev. 2 (2004); David Sleeth-Keppler, *Taking the High (or Low) Road: A Quantifier Priming Perspective on Basic Anchoring Effects*, 153 J. Soc. Psychol. 424 (2013); Nicole M. Long et al., *Memory and Attention*, *in* 1 Stevens’ Handbook of Experimental Psychology and Cognitive Neuroscience 285 (John T. Wixted et al. eds., 4th ed. 2017).

27 See discussion accompanying notes 180–181.

28 See Henry L. Roediger III et al., *The Curious Complexity between Confidence and Accuracy in Reports from Memory*, *in* Memory and Law, *supra* note 6, at 91.

29 Elizabeth Loftus has written in great detail about the effects of false memories in legal proceedings. See Daniel M. Bernstein & Elizabeth F. Loftus, *How to Tell if a Particular Memory is True or False,* 4 Perspectives on Psychol. Sci. 370 (2009) (detailing measures lawyers take to determine whether an individual can be believed); Ira E. Hyman & Elizabeth F. Loftus, *Errors in Autobiographical Memory,* 18 Clinical Psychol. Rev. 933 (1998) (writing how memory is constructive and thus errors are likely to occur); Elizabeth F. Loftus, *Planting Misinformation in the Human Mind: A 30-year Investigation of the Malleability of Memory*, Learning & Memory 361 (2005) (detailing how misleading information can affect one’s memory in damaging ways); see also Schacter, *supra* note 25 (describing ‘sins’ of memory—‘transience, absent-mindless, blocking, misattribution, suggestibility, bias, and persistence’—that lead individuals to confidently claim they have experienced events that never occurred.)

30 See generally Endel Tulving, *Episodic Memory: From Mind to Brain*, 53 Ann. Rev. Psychol. 1 (2002).

31 See Bruno Verschuere & Bennett Kleinberg, *Assessing Autobiographical Memory: The Web-based Autobiographical Implicit Association Test*, 25 Memory 520, 527–28 (2016); see also J. Peter Rosenfeld et al., *Instructions to Suppress Semantic Memory Enhances or Has No Effect on P300 in a Concealed Information Test (CIT)*, 113 Int. J. Psychol. 29, 30 (2017).

32 Louis Renoult et al., *Personal Semantics: At the Crossroads of Semantic and Episodic Memory*, 16 Trends Cognitive Sci. 550, 550–56 (2012).

33
*Id.*

34 Consider, as an illustration of how it can be difficult to make inferences about an underlying cognitive process based on behavior, this example from Renoult et al., *id.* at 554, about how single episodes can later be retrieved as autobiographical facts: ‘I may know what occurred during my brother’s speech at my wedding, without having a perceptually rich, first-person re-experiencing of it. In other words, this single event could be retrieved with noetic awareness, that is, the type of awareness that is associated with knowing about the world and retrieving semantic knowledge, rather than with autonoetic awareness, that is, the type of awareness associated with subjectively re-experiencing events from [episodic memory.]’ Memory researchers attempt to overcome this difficulty with the ‘remember/know procedure, in which participants report on the type of awareness that they have experienced during retrieval’, along with other methods. *Id.*

35 A recent study demonstrated that fMRI with machine-learning algorithm analysis could predict with a high degree of accuracy whether a study subject was in a ‘knowing’ or ‘reckless’ state of mind ‘at the time’ they made a decision whether to carry a suitcase potentially or actually containing contraband through a security checkpoint that could potentially be searched. Iris Vilares, Michael Wesley, Woo-Young Ahn, Richard J. Bonnie, Morris B. Hoffman, Owen D. Jones, Stephen J. Morse, Gideon Yaffe, Terry Lohrenz, & Read Montague, *Predicting the Knowledge-Recklessness Distinction in the Human Brain,* 14 Proc. Nat’l Acad. Sci. 3222 (2017). See also Owen D. Jones, Gideon Yaffe, & Read Montague, *Detecting* Mens Rea *in the Brain*, 169 U. Pennsylvania L.R. (forthcoming 2020), writing ‘by combining fMRI brain-imaging techniques with a machine learning algorithm we were able to distinguish among guilty minds’. Importantly for this analysis, the proof of concept is that the culpable mental states of ‘knowing’ and ‘reckless’ are neurally and psychologically distinguishable, but not that they could be retroactively distinguished. ‘Our team’s neuroscientific techniques can discover brain-based differences between mental states that exist at the time of scanning, *not at some prior time*… our current experiment has implications for criminal justice policy, but not for forensic evaluation of individual defendants’ *Id*.

36 Brown and Murphy, *supra* note 5, at 1130–31, 1187–88.

37 Here we use ‘biological’ to encompass psychological phenomena as well, referring to mechanisms intrinsic to the person, rather than to the tools being used to assess the person.

38 See eg Overview, Farwell Brain Fingerprinting—Dr. Larry Farwell, https://larryfarwell.com/brain-fingerprinting-overview-dr-larry-farwell-dr-lawrence-farwell.html (accessed Mar. 12, 2020) and *iCognative Technology*, Brainwave Science, http://brainwavescience.com/icognative/ (accessed March 12, 2020). Farwell now complains that Brainwave Science stole his technology.

39 Courts in India and police services in Singapore have employed BEOS technology. David Cox, ‘Can Your Brain Reveal You are a Liar?’, BBC Future (Jan. 25, 2016).

40 See generally Meegan, *supra* note 14 (concluding that EEG-based memory detection techniques have forensic potential but require additional research and considerations of legal admissibility and ethical issues.)

41 Lawrence Farwell, an American scientist, markets his ‘brain fingerprinting’ technology as ‘admissible in court’ and sells it to international law enforcement departments. See Larry Farwell Brain Fingerprinting Laboratories, https://larryfarwell.com/brain-fingerprinting-laboratories-inc.html (accessed Mar. 20, 2020). Farwell was an early participant in peer reviewed research on memory detection, see Lawrence A. Farwell & Emanuel Donchin, *The Truth Will Out: Interrogative Polygraphy (“Lie Detection”) with Event-Related Brain Potentials*, 28 Psychophysiology 531 (1991); but his more recent and product-focused work (reporting suspicious 0 per cent error rates) has been critiqued by other researchers,. *See* J. Peter Rosenfeld, *‘Brain Fingerprinting’: A Critical Analysis*, 4 Sci. Rev. Mental Health Prac. 20 (2005) for a critique of the vague definition of Farwell’s patented P300-MERMER response. In 2012, Farwell published *Brain Fingerprinting: A Comprehensive Tutorial Review of Detection of Concealed Information with Event-Related Brain Potentials,* 6 Cognitive Neurodynamics 115 (2012). Other researchers responded forcefully, arguing that Farwell’s ‘tutorial’ is ‘misleading and misrepresents the scientific status of brain fingerprinting technology’, And noting a paucity of peer-reviewed data and selection bias in Farwell’s reporting. See Ewout H. Meijer et al., *A Comment on Farwell (2012): Brain Fingerprinting: A Comprehensive Tutorial Review of Detection of Concealed Information with Event-Related Brain Potentials,* 7 Cognitive Neurodynamics 155 (2013). In 2013, Farwell and colleagues published a study comparing error rates and accuracy in four separate field studies, once again reporting an unbelievable 100 per cent accuracy, 0 per cent error rate, no false positives or false negatives, and no vulnerability to countermeasures for studies using P300-MERMER. Lawrence A. Farwell et al., *Brain Fingerprinting Field Studies Comparing P300-MERMER and P300 Brainwave Responses in the Detection of Concealed Information,* 7 Cognitive Neurodynamics 263 (2013).

42 ‘Meaningful’ stimuli may seem a vague term. This is deliberate, because the psychological characteristics of such ‘meaningful’ stimuli that trigger a particular brain response may be ‘meaningful’ for different reasons. For example, it may be inaccurate to say something is ‘remembered’ just because a subject’s brain gives a characteristic response. The same P300 ERP response may be elicited by stimuli that are remembered, recognized, familiar, or even simply highly salient to a subject. For example, a gun encountered amidst a series of innocuous object stimuli would evoke a P300, but a gun encountered amidst an array of different weapons (e.g., dagger, switchblade, crow bar, etc.) would not automatically evoke a P300. It would only evoke a P300 if it was meaningful to the subject. But, this could be because the subject is a gun enthusiast, or because s/he thinks that the gun is the ‘guilty knowledge’ stimulus the investigators are interested in, even if s/he did not commit the crime. A more effective assessment using the P300 would be to flash a series of different guns, with only one being the gun actually found at the crime scene or known to be the murder weapon.

43 Rosenfeld describes the P300 as ‘a cortical signal of the recognition of meaningful information’. See J. Peter Rosenfeld, *P300 in Detecting Concealed Information*, *in* Memory Detection, *supra* note 6, at 63.

44 Meegan, *supra* note 14, at 12; *see generally* John Polich & Albert Kok, *Cognitive and Biological Determinants of P300: An Integrative Review*, 41 Biological Psychol. 103 (1995); see also Rosenfeld, *supra* note 43, at 64. A separate ERP response, an anterior signal the labeled N200 (or sometimes N2), was at one point suggested to respond selectively to probes v. irrelevants and thought to index cognitive control processes when lying about probes. Matthias Gamer & Stefan Berti, *Task Relevance and Recognition of Concealed Information have Different Influences on Electrodermal Activity and Event-Related Brain Potentials*, 47 Psychophysiology 355 (2010). But a subsequent study found that this effect was likely due to an experiment design flaw, and that the N2 differences were the result of stimulus differences rather than concealed information differences. Giorgio Ganis et al., *Is Anterior N2 Enhancement a Reliable Electrophysiological Index of Concealed Information?*, 143 Neuroimage 152 (2016).

45 Rosenfeld, *supra* note 43, at 64.

46 Meegan, *supra* note 14, at 12.

47 Running a single individual through multiple testing runs to obtain enough data for averages of different trial types is problematic, especially infrequent trial types such as probes and irrelevants. Repeating a test can create habituation effects that may confound probe effects, and previously irrelevant stimuli could become relevant through repetition. Rosenfeld, *supra* note 43, at 69. A statistical analytic method called ‘bootstrapping’ is used to assess differences between stimuli types for a single individual subject in one run. Bootstrapped averages are created by repeatedly resampling from the set of traces for a particular probe (or irrelevant) ‘with replacement’, meaning the same trace could be sampled more than once. For each resampled set, an average is created. The process is iterated many times to create a distribution of averages. Then, a *t*-test on the resampled averages is more sensitive than a test on single ERP traces, which have a large amount of noise. See *Id.* This technique effectively treats the raw data, which is in reality a sample of the ‘population’ of brain traces for a given individual, as the ‘population’ to be analyzed by resampling. This technique was introduced into the EEG memory detection literature by Farwell & Donchin, *supra* note 41, in 1991. Rosenfeld’s research group ‘typically require[s] that there must be at least .9 (90 per cent) level of statistical confidence that the average probe P300 is greater than the average of all irrelevant P300s before concluding that a subject recognizes concealed information germane to a crime’, but caution that ‘until a finalized and definitive P300-based test is developed and… optimized for maximum discrimination efficiency and accuracy, as confirmed in representative populations, one cannot arbitrarily set a bootstrap diagnostic criterion at some level for use in all future studies’. Rosenfeld, *supra* note 8, at 119.

48 Matthias Gamer et al., *Combining Physiological Measures in the Detection of Concealed Information*, 95 Physiology & Behav. 333, 333 (2007). But see recent evidence that single trial EEG data can be used to classify memory retrieval outcomes on individual trials with modest yet above-chance accuracy (56–61 per cent correct, with 50 per cent as chance). Eunho Noh et al., *Single-Trial EEG Analysis Predicts Memory Retrieval and Reveals Source-Dependent Differences*, 12 Frontiers Hum. Neurosci., Article 258 (July 2018).

49 For any version of the CIT, multiple choice options are offered. On any given trial, the likelihood of a false positive is determined by the number of options: 25 per cent if there are four choices, 20 per cent if there are five choices. By adding more questions with independent items of information, the likelihood of an overall false positive result decreases, by virtue of multiplying the likelihood of each independent trial. Test designers must choose a threshold for how many ‘hits’ across multiple trials would lead to a ‘guilty’ determination, accounting for the likelihood of false positives and a normatively determined acceptable threshold of error. Rosenfeld et al. tout this as an advantage of the CIT: ‘The greater the number of independent items, the greater the protection against false positive diagnoses.’ Rosenfeld et al., *supra* note 8, at 119. But requiring more independent items to be tested to lower the false positive probability to an acceptable threshold may prove extremely difficult in practice, where the set of ‘probes’ known only to a guilty subject and investigators may be limited.

50 An operating assumption behind brain-based memory detection research is that techniques may access psychological processes of recognition that may be ‘sufficiently automatic as to be relatively invulnerable to countermeasures’. Meegan, *supra* note 14, at 18. Meegan examined the potential for EEG-based studies to be vulnerable to countermeasures under the rubric of ‘retrieval automaticity’. *Id.* at 10–13. He described two strategies that could be used by a ‘guilty’ subject attempting to beat a P300-based test: (i) producing an irrelevant-like P300 for probes (creating false negatives), or (ii) producing a probe-like P300 for irrelevants (creating false positives). Meegan identified the former strategy as particularly challenging and not yet supported by existing evidence, given that it is ‘difficult to treat something meaningful as meaningless’. *Id.* at 13. The latter strategy has been examined, starting in 2004 when Rosenfeld et al. trained ‘guilty’ participants (who had committed a mock crime) to make irrelevants seem like task-relevant stimuli by making a distinct covert physical response (such as pressing a particular finger into their leg, or wiggling a toe) to different categories of irrelevants, resulting in a P300 similar to that of probes. J. Peter Rosenfeld et al., *Simple, Effective Countermeasures to P300-based Tests of Detection of Concealed Information*, 41 Psychophysiology 205 (2004).

51 J. Peter Rosenfeld et al., *The Complex Trial Protocol (CTP): A New, Countermeasure-Resistant, Accurate, P300-based Method for Detection of Concealed Information*, 45 Psychophysiology 906, 906–07 (2008).

52
*Id.* See also J. Peter Rosenfeld & Henry T. Greely*, Deception, Detection of, P300 Event-Related Potential (ERP),* in The Wiley Encyclopedia of Forensic Science (Allan Jamieson & Andre A. Moenssens eds., 2012).

53 Rosenfeld et al., *supra* note 50, at 208–09 (describing wiggling various toes or pressing various fingers); Ralf Mertens & John JB Allen, *The Role of Psychophysiology in Forensic Assessments: Deception Detection, ERPs, and Virtual Reality Mock Crime Scenarios*, 45 Psychophysiology 286 (2008).

54 Alexander Sokolovsky et al., *A Novel Countermeasure Against the Reaction Time Index of Countermeasure Use in the P300-based Complex Trial Protocol for Detection of Concealed Information*, 81 Int. J. Psychophysiology 60, 61 (2011).

55 Rosenfeld et al., *supra* note 51, at 907–08; Michael R. Winograd & J. Peter Rosenfeld, *Mock Crime Application of the Complex Trial Protocol (CTP) P300-Based Concealed Information Test*, 48 Psychophysiology 155 (2011).

56 Sokolovsky et al., *supra* note 54.

57 Zara M. Bergstrom et al., *Intentional Retrieval Suppression Can Conceal Guilty Knowledge in ERP Memory Detection Tests*, 94 Biological Psychol. 1 (2013). Bergstrom et al. used a three stimulus protocol; ‘guilty’ subjects were assigned to be cooperative and rehearse their response knowledge upon cue presentation, or be uncooperative and try to suppress knowledge coming to mind in response to the cue. *Id.* at 3–4. The P300 response was smaller in the latter group, but evidence for active suppression is confounded by the fact that the first group ‘actively rehearsed’, as opposed to being passively knowledgeable about their guilty knowledge. *Id.* at 4–10. In 2015, Hu et al. added a ‘simply knowledgeable’ group in a mock crime scenario tested with the CTP and found differences in the P300 between the ‘simply knowledgeable’ guilty group and the actively suppressing guilty group. Xiaoqing Hu, et al., *Suppressing Unwanted Autobiographical Memories Reduces Their Automatic Influences: Evidence From Electrophysiology and an Implicit Autobiographical Memory Test*, 26 Psychol. Sci. 1098, 1099–1104 (2015). But the differences were based on a particular method of measuring the P300 (baseline to peak), while a different method (peak to peak) is substantially more accurate in discriminating knowledgeable and naive subjects. See also Rosenfeld et al., *supra* note 31.

58 Anne C. Ward & J. Peter Rosenfeld, *Attempts to Suppress Episodic Memories Fail but Do Produce Demand: Evidence from the P300-Based Complex Trial Protocol and an Implicit Memory Test*, 42 Applied Psychophysiology Biofeedback 13, 24 (2014) (reporting that, with a single discrete change to the CTP used by the 2015 paper—changing the ratio of target to non-target items to make the task slightly easier—attempts to suppress reduction of P300 to probe responses did not give significant differences as compared to a ‘simply guilty’ control group).

59 See Rosenfeld et al., *supra* note 31, at 30.

60 Rosenfeld reported on an experiment where subjects pretended to steal 1 of 10 items from a box, and then were shown the names of all 10 items one at a time. The items that subjects pretended to steal evoked target-like P300s, whereas the non-stolen items did not. J. Peter Rosenfeld et al., *Late Vertex Positivity in Event-Related Potentials as a Guilty Knowledge Indicator: A New Method of Lie Detection*, 34 Int’l L.J. Neurosci. 125 (1987). Farwell and Donchin reported on two experiments, the first a mock espionage scenario in which, after extensive training, briefcases were transferred to confederates in code name operations. The second experiment tested four subjects who were admittedly guilty of minor transgressions on campus. Farwell & Donchin, *supra* note 41. Winograd and Rosenfeld conducted a mock crime experiment where some subjects were given instructions to steal a ‘ring’, whereas others were told to steal an ‘item’, finding that having knowledge of the crime created a high rate of false positives. Winograd & Rosenfeld, *infra* note 63. Hu et al. tested the effect of a delayed test, determining that subjects tested a month after being told to ‘steal’ an exam were detected at a similar rate as those who were tested immediately after the crime. Hu et al., *infra* note 65.

61 John B. Meixner & J. Peter Rosenfeld, *Detecting Knowledge of Incidentally Acquired, Real-World Memories Using a P300-Based Concealed-Information Test,* 25 Psychol. Sci. 1 (2014).

62
*Id.* at 2–4.

63 Michael R. Winograd & J. Peter Rosenfeld, *The Impact of Prior Knowledge from Participant Instructions in a Mock Crime P300 Concealed Information Test*, 94 Int’l J. Psychophysiology 473, 473 (2014); see also Winograd & Rosenfeld, *supra* note 55.

64 Rosenfeld et al., *supra* note 8, at 122.

65 Xiaoqing Hu et al., *Combating Automatic Autobiographical Associations: The Effect of Instruction and Training in Strategically Concealing Information in the Autobiographical Implicit Association Test*, 23 Psychol. Sci. 1079, 1080 (2012).

66 Pranav Misra et al., *Minimal Memory for Details in Real Life Events*, 8 Sci. Rep., Article 16,701 (2018).

67 Rosenfeld, *supra* note 8, at 125.

68 Xiaoqing Hu et al., *N200 and P300 as Orthogonal and Integrable Indicators of Distinct Awareness and Recognition Processes in Memory Detection*, 50 Psychophysiology 454, 462–65 (2013). Researchers have questioned whether supplementing P300-based protocols with feedback about a subject’s responses could improve the test’s detection efficiency. In a recent study using a mock-crime scenario and the CTP, providing non-veridical feedback directing deceptive subjects’ attention to the probe resulted in a larger P300 signal (probe-irrelevant difference) than did providing generic feedback regarding test performance. The study’s authors refer to the probed material as ‘incidentally acquired’, apparently meaning that the identity of the to-be-stolen item (a ring) was only acquired if the subject actually stole the ring, rather than via instructions to steal it (or not to steal it, for ‘innocent’ subjects). In the ‘high awareness’ condition, where feedback told the subject ‘based on your brainwaves, it seems that you are following task instructions well’, the P300 could effectively differentiate guilty from innocent participants. But in the ‘low awareness’ condition, where subjects received feedback that ‘based on your brainwaves, it seems you are following task instructions well’, detecting guilty subjects was not significantly different from chance.

69
*Id.* They also reported a frontally centrally distributed N200 ERP response to probes in the ‘high awareness’ group of guilty participants who had stolen the ring and were told that their brainwaves identified something as important, as compared to innocent or ‘low awareness’ groups (who were told their brainwaves indicated they followed directions well). Hu et al. theorize that processes other than recognition may be mechanisms by which memory detection works and that combining them may improve detection efficiency. *Id.* Further research on the mechanisms that elicit the N200 is needed.

70 Researchers have attempted to combine P300-based tests with autobiographical versions of the Implicit Association Test (aIAT). See Hu et al., *supra* note 65.

71 See generally Richard A. Carlson & Don E. Dulany, *Conscious Attention and Abstraction in Concept Learning*, 11 J. Exp. Psychol. 45 (1985); Eric Eich, *Memory for Unattended Events: Remembering With and Without Awareness*, 12 Memory & Cognition 105 (1984); Ronald T. Kellogg & Ruth S. Dare, *Explicit Memory for Unattended Information*, 27 Bull. Psychonomic Soc’y 409 (1989).

72 Consider a home break-in scenario, where perpetrators grab items perceived to have value without closely examining them, such as emptying a jewelry box. In such a scenario, a victim’s inventory of missing items may have little use in investigating a suspect, who may have never encoded the Art Deco earrings or mother-of-pearl brooch.

73 For a recent review, see Stephanie A. Gagnon & Anthony D. Wagner, *Acute stress and episodic memory retrieval: neurobiological mechanisms and behavioral consequences*, 1369 Ann. N.Y. Acad. Sci. 55–75 (2016). In general, acute stress can enhance encoding and consolidation of episodic memory, particularly for emotional events. *Id.* at 59. But the research findings are complex and nuanced, and not necessarily linear in terms of the relationship between stress and encoding, consolidation, recognition, and recall, and there are conflicting findings as to whether stress differentially affects emotional or neutral stimuli.

74
*Id.* at 60. However, there are differential impacts depending on the retrieval task, and it may be the case that probed memory detection paradigms that present information to be recognized would be least affected by stress at the time of retrieval: ‘Stress-related impairments of episodic retrieval tend to be greater on tests requiring free recall relative to cued recall and cued recall relative to recognition’. See also Stephanie A. Gagnon, Michael L. Waskom, Thackery I. Brown & Anthony D. Wagner, *Stress impairs episodic retrieval by disrupting hippocampal and cortical mechanisms of remembering*, 29 Cerebral Cortex 2947–2964 (2019) (Assessing recollection in a cued recall task under threat of electric shock, using fMRI with multivariate pattern analysis: ‘When stressed, people are less likely to recollect specific details about past events, and, even when expressing high confidence in what was remembered, they are more likely to produce less accurate memories’.).

75 Brown & Murphy, *supra* note 5.

76 Brain Research through Advancing Innovative Neurotechnologies (BRAIN) Working Group, Brain 2025: A Scientific Vision 41–42 (2014), https://braininitiative.nih.gov/strategic-planning/brain-2025-report.

77 See Nicco Reggente et al., *Enhancing the Ecological Validity of fMRI Memory Research Using Virtual Reality*, 12 Frontiers Neurosci., Article 408 (June 2018).

78 For an accessible review of these methodological approaches, see Joey Ka-Yee Essoe & Jesse Rissman. *Applications of Functional MRI in Memory Research*, in Handbook of Research Methods in Human Memory 397–427 (H. Otani & B.L. Schwartz, eds., 2018).

79 This literature has been comprehensively reviewed in Nancy A. Dennis et al., *Functional Neuroimaging of False Memories*, in The Wiley Handbook on The Cognitive Neuroscience of Memory 150 (Donna Rose Addis et al. eds., 1st ed. 2015); see also Daniel L. Schacter et al., *Neuroimaging of True, False, and Imaginary Memories*, in Memory and Law, *supra* note 6, at 233.

80 Dennis et al., *supra* note 79, at 152.

81
*Id.* at 154–55.

82
*Id.* at 157–59.

83 Avi Mendelsohn et al., *Signature of Memory: Brain Coactivations During Retrieval Distinguish Correct from Incorrect Recollection*, 4 Frontiers Behav. Neurosci., Article 18 (April 2010) at 1.

84 Jesse Rissman & Anthony D. Wagner, *Distributed representations in memory: insights from functional brain imaging*, 63 Ann. Rev. Psychol. 101–28 (2012).

85 See generally James V. Haxby, *Multivariate Pattern Analysis of fMRI: The Early Beginnings*, 62 Neuroimage 852 (2012).

86 Jesse Rissman et al., *Detecting Individual Memories Through the Neural Decoding of Memory States and Past Experience*, 107 Proceedings Nat’l Acad. Sci. 9849, 9852 (2010).

87 The classifiers were also highly accurate in determining ‘whether participants’ recognition experiences were associated with subjective reports of recollection, a strong sense of familiarity, or only weak familiarity, with the discrimination between recollection and strong familiarity being superior to that between strong v. weak familiarity’. *Id.* And in a second experiment that probed implicit memory for oldness or newness, participants were required to study faces before scanning and then, in the scanner, make male/female judgments rather than old/new judgments. In this situation, the classification methods were ‘not capable of robustly decoding the OLD/NEW status of faces encountered during the Implicit Recognition Task’, leading the researchers to conclude that ‘a neural signature of past experience could not be reliably decoded during implicit recognition’. *Id.* at 9852–53. This suggests that encoding environment matters significantly for the detectability of details of life experiences that may only be incidentally encoded, rather than the focus of attention. Such a finding has implications for stimulus selection in any forensic context—perhaps only a murderer would know that the victim was found on a paisley-pattered couch, but if the murderer paid no attention to the couch pattern, such a unique detail may be only incidentally encoded and thus cannot be reliably detected. But see Brice A. Kuhl et al., *Dissociable Neural Mechanisms for Goal-Directed Versus Incidental Memory Reactivation*, 33 J. Neurosci. 16099, 16099–109 (2013) (finding that mnemonic information could be decoded even when participants are not instructed to attend to that information).

88 Rissman et al., *supra* note 86, at 9852–53.

89 Melina R. Uncapher et al., *Goal-Direct Modulation of Neural Memory Patterns: Implications for fMRI-Based Memory Detection*, 35 J. Neurosci. 8531 (2015).

90
*Id.* at 8537–39.

91
*Id.* at 8545.

92 Rissman et al., *supra* note 86, at 9851–52; see also 10 (Figure S5) in *Supporting Information for Rissman et al.*, PNAS, https://www.pnas.org/content/pnas/suppl/2010/04/30/1001028107.DCSupplemental/pnas.201001028SI.pdf (accessed Apr. 16, 2020) (hereinafter Supporting Information).

93 The authors discuss in the Supporting Information: ‘Pattern classification analyses were implemented in MATLAB using routines from the Princeton MVPA Toolbox and custom code…. A variety of machine learning algorithms have been successfully used to decode cognitive states from fMRI data. Here, we explored several algorithms, including two-layer back-propagation neural networks, linear support vector machines, and regularized logistic regression. Although all three performed well, we found that RLR generally outperformed the other techniques, if only by a small amount…. Thus, we elected to use RLR for all classification analyses reported in the manuscript’. *Id.* at 2, 4. The differences between these types of machine learning algorithms matter greatly for how extensively their credibility can be tested—or, conversely, how much like a true ‘black box’ they are such that even the experts employing them are unable to truly explain the analytical steps taken to reach a particular inference. See text accompanying notes 182–205, *infra*. Researchers do select cutoff thresholds, or decision boundaries to adjust the sensitivity or specificity of labeling examples of Class A or Class B and tolerances for false positives and false negatives.

94 Rissman et al., *supra* note 86 at 9853 (‘Although the predictive value of this classification was relatively poor (mean AUC = 0.59), the modest success of this classifier suggests that the neural signatures of true and false recognition are at least sometimes distinguishable’.).

95 For a review of recent memory work using camera technology to extend research findings of lab-based memories, see Tiffany Chow & Jesse Rissman, *Neurocognitive Mechanisms of Real-World Autobiographical Memory Retrieval: Insights from Studies Using Wearable Camera Technology*, 1396 Annals N.Y. Acad. Sci. 202 (2017). See also Peggy L. St. Jacques & Felipe De Brigard, *Neural Correlates of Autobiographical Memory: Methodological Considerations*, in The Wiley Handbook on The Cognitive Neuroscience of Memory, *supra* note 79, at 265.

96 Jesse Rissman et al., *Decoding fMRI Signatures of Real-world Autobiographical Memory Retrieval*, 28 J. Cognitive Neurosci. 604, 606–07 (2016).

97
*Id.* at 610–14. See also Chen et al., *supra* note 23 (reporting different patterns of network activation for recollection of studied pictures versus autobiographical events).

98 Rissman et al., *supra* note 96, at 615–17.

99 F. Milton, Nils Muhlert, C. R. Butler, A. Smith, A. Benattayallah & Adam Z. Zeman, *An fMRI study of long-term everyday memory using SenseCam*, 19 Memory 733–744 (2011).

100
*Id.* at 616–17.

101 Judith Peth et al., *Memory Detection using fMRI—Does the Encoding Context Matter?* 113 NeuroImage 164, 165–66 (2015).

102
*Id.* at 168–72. The authors also performed univariate analyses, reporting that those results ‘further support the assumption that memory and not deception is the key mechanism for successful detection of information with the CIT…. Taken together, univariate fMRI analyses indicate that the CIT can be primarily used to detect the presence of critical information but does not directly allow for determining the source of knowledge’.

103 Winograd & Rosenfeld, *supra* note 63.

104 See Chow et al., *supra* note 23, at 121 (‘[i]n some ways, the term “laboratory-based” may be a misnomer, since there is nothing intrinsically special about encoding information while participating in a psychology experiment versus encoding information outside of the lab (ie, in “real life”). Thus, the divergent patterns of brain activation observed during the retrieval of these kinds of memories may be driven to a large degree by differences in the mnemonic processes evoked (eg recognition as based on either contextual recollection or item familiarity), methodology (eg perceptual qualities of the stimuli used to probe memories), or even characteristics of the tested memories themselves (eg personal relevance or temporal remoteness)’.)

105
*Id.* at 112.

106
*See* Brown & Murphy, *supra* note 5.

107 See Maxwell A. Bertolero & Danielle A. Bassett, *Deep Neural Networks Carve the Brain at Its Joints*, pre-print (revised Sep. 9, 2020), https://arxiv.org/abs/2002.08891v2.

108 See eg John B. Meixner & J. Peter Rosenfeld, *A Mock Terrorism Application of the P300-Based Concealed Information Test*, 48 Psychophysiology 149 (2011) (using the CIT in a mock terrorism scenario to effectively detect criminal information).

109 Probably not, according to classic findings by Christianson and colleagues that memories for events during increased arousal exhibit a concentration on central details with reduced recall of peripheral details. Sven Åke Christianson et al., *Eye Fixations and Memory for Emotional Events*, 17 J. Exp. Psychol. 693, 695–700 (1991).

110 This is the attribute of specificity discussed by Meegan, *supra* note 14.

111 See eg Bernstein & Loftus, *supra* note 29, at 373 (concluding that ‘[i]n essence, all memory is false to some degree. Memory is inherently a reconstructive process, whereby we piece together the past to form a coherent narrative that becomes our autobiography’); see also Demis Hassabis & Eleanor A. Maguire, *The Construction System of the* Brain, 364 Phil. Transactions Royal Soc’y B 1263 (2009).

112 See eg Memory and Law, *supra* note 6.

113 Hearsay rules are predicated, in part, on the fallibility of a human’s memory. See eg Anders Sandberg et al., *The Memory of Jurors: Enhancing Trial Performance, in* Memory and Law, *supra* note 6, at 213; see also Daniel L. Schacter & Elizabeth Loftus, *Memory and Law: What Can Cognitive Neuroscience Contribute?*, 16 Nature Neurosci. 119 (2013); Joyce W. Lacy & Craig E.L. Stark, *The Neuroscience of Memory: Implications for the Courtroom,* 14 Nature Rev. Neurosci. 649 (2013).

114 Fed. R. Evid. 701, Fed. R. Evid. 702, Daubert v. Merrell Dow Pharmaceuticals, Inc., 509 U.S. 579 (1993).

115 Fed. R. Evid. 401, Fed. R. Evid. 402.

116 For example, if the witness were to testify as that ‘it was sunny at the time I saw the accident’, and the corroborating weather report were available that day, there would be no practical need to validate or verify the witness’s memory as to the weather.

117 Fed. R. Evid. 403.

118 See Brown & Murphy, *supra* note 5.

119 Of course, there may be cases where such a device would be helpful, such as concealed knowledge cases where detecting a memory would suggest the accuracy of the facts remembered because it would be an incredible coincidence to have formed a false, but subjectively believed, memory about the concealed facts. This, however, is an issue of ‘corroboration’—to be certain they are in such a situation, investigators must already know something about the ground truth, and, again, the person concealing the knowledge they possess is essentially deceiving investigators by declining to disclose it, so we are back in the realm of lie detection. Alternatively, Bayesians would point out that if one of three possibilities—true memory, false memory, and lie—can be eliminated (the lie), it is helpful for a decision maker to eliminate that possibility. Epistemologically, this may improve accuracy in decision-making, but raises the same process concerns as lie detection devices.

120 We thank Jason Rantanen for the example.

121 Yuhwa Han, *Deception Detection Techniques Using Polygraph in Trials: Current Status and Social Scientific Evidence*, 8 Contemp. Readings L. & Soc. J. 115 (2016)

122 See Fed. R. Evid. 702(a).

123 The ‘statement’ would be the report generated by the putative memory detection device, assuming such existed. The declarant would be the person running the examination, as a report produced exclusively by machine cannot be hearsay, as a machine cannot be a declarant. But, as seen above, any forensic memory detection test would require specific examiner decision-making and input as to stimuli, task design, and any programming or instructions provided to the examinee and/or device. The forensic memory examiner would certainly be the declarant for any resulting report produced. Some jurisdictions have evaded the ‘machine as declarant’ problem for highly standardized tests by making printouts that record the results of certain mechanical tests (eg blood or breath alcohol) admissible by statute. Technically, such laws create an exception to the hearsay rule, though they often treated as authentication issues. Arguably, whether the person being tested would also be considered a ‘declarant’, and whether such evidence would present double hearsay problems, may depend upon whether the person submitting to the examination does so intending to assert the contents of their memory as verifiable. This would generally be true for a self-interested defendant who solicited a scan to prove an alibi, or a lack of crime-relevant knowledge. But it would arguably not be true for a suspect compelled to submit to a test for which affirmative verbal or behavioral responses were not required, such as in the BEOS paradigm. Such compulsions would raise practical issues, but also legal privacy concerns and potentially constitutional concerns about self-incrimination. See eg Nita A. Farahany, *Searching Secrets*, 160 Penn. L. Rev. 1239 (2012). See also Bard, *supra* note 5 (outlining Fourth, Fifth, and Sixth Amendment concerns about neuroimaging-obtained information).

124 Past statements about a present state of mind or future intention are (perhaps nonsensically) considered an exception to the rule against hearsay precisely because they lack concerns about faulty memory or faulty perception. See eg Fed. R. Evid. 803(3); Mutual Life Insurance Co. v. Hillmon, 145 U.S. 285 (1892); John MacArthur Maguire, *The Hillmon Case—Thirty-Three Years After*, 38 Harv. L. Rev. 709 (1925).

125 See *supra note* 123. Several circuits have held that machine statements are not hearsay. *See* United States v. Lizarraga-Tirado, 789 F.3d 1107, 1110 (9th Cir. 2015); United States v. Lamons, 532 F.3d 1251, 1263 (11th Cir. 2008); United States v. Moon, 512 F.3d 359, 362 (7th Cir. 2008); United States v. Washington, 498 F.3d 225, 230 (4th Cir. 2007); United States v. Hamilton, 413 F.3d 1138, 1142 (10th Cir. 2005); United States v. Khorozian, 333 F.3d 498, 506 (3d Cir. 2003).

126 Fed. R. Evid. 705.

127 See eg Archie Alexander, *Functional Magnetic Resonance Imaging Lie Detection: Is a Brainstorm Heading toward the Gatekeeper?* 7 Hous. J. Health L. & Pol’y 1 (2007); Benjamin Holley, *It’s All in Your Head: Neurotechnological Lie Detection and the Fourth and Fifth Amendments*, 28 Dev. Mental Health L. 1 (2009); Leo Kittay, *Admissibility of fMRI Lie Detection: The Cultural Bias Against “Mind Reading” Devices*, 72 Brook. L. Rev. 1351 (2007); Daniel D. Langleben & Jane Campbell Moriarty, *Using Brain Imaging for Lie Detection: Where Science, Law, and Policy Collide,* 19 Psychol., Pub. Pol’y, L. 222, 231 (2012) (noting that the ‘most important missing piece in the puzzle is *Daubert*’s “known error rate” standard. Determining the error rates for fMRI-based lie detection requires validation of the method in settings convincingly approximating the real-life situations in which legally significant deception takes place, in terms of the risk–benefit ration, relevant demographics, and the prevalence of the behavior in question’.); Joëlle Anne Moreno, *The Future of Neuroimaged Lie Detection and the Law*, 42 Akron L. rev. 717 (2009) (finding that a fMRI-detected increase in blood flow does not necessarily indicate lying; there are other emotional states that may cause the prefrontal cortex to activate); Jane Campbell Moriarty, *Visions of Deception: Neuroimages and the Search for Truth*, 42 Akron L. Rev. 739 (2009); Sean A. Spence & Catherine J. Kaylor-Hughes, *Looking for Truth and Finding Lies: The Prospects for a Nascent Neuroimaging of Deception*, 14 Neurocase 68 (2008); Sean A. Spence, *Playing Devil’s Advocate: The Case Against fMRI Lie Detection*, 13 Legal & Criminological Psychol. 11 (2008) (finding that most fMRI lie detection research lacks construct and external validity); Zachary E. Shapiro, Note, *Truth, Deceit, and Neuroimaging: Can Functional Resonance Imaging Serve as a Technology-Based Method of Lie Detection?*, 29 Harv. J. L. & Tech. 527 (2016). *But see* Daniel D. Langleben et al., *Brain Imaging of Deception,* in Neuroimaging in Forensic Psychiatry: From the Clinic to the Courtroom (Joseph R. Simpson ed., 2012); Frederick Shauer, *Can Bad Science Be Good Evidence—Neuroscience, Lie Detection, and Beyond*, 95 Cornell L. Rev. 1191 (2010); see also David H. Kaye et al., *How Good is Good Enough: Expert Evidence Under Daubert and Kumho*, 50 Case W. Res. L. Rev. 645 (2000).

128 United States v. Semrau, 693 F.3d 510 (6th Cir. 2012); Memorandum Opinion and Order at 5–6, Maryland v. Smith, No. 106589C, (Montgomery Cty, MD, Oct. 3, 2012); Wilson v. Corestaff Services, L.P., 900 N.Y.S.2d 639 (N.Y. Sup. Ct. 2010).

129 Meixner, Jr., *supra* note 6; see also Meixner, *supra* note 7; Rosenfeld et al., *supra* note 8.

130 A slightly different analysis would follow if a testifying expert such as a treating psychiatrist or psychologist claimed to ‘reasonably rely’ upon memory detection technology in developing their overall assessment of a subject’s mental state. Fed. R. Evid. 703. For this route to lead to admissibility, memory detection evidence would likely need to be commonly used by such experts in forming their opinion on the subject. This does not seem to be a ‘back door’ around *Daubert*, as it is unlikely that expert reliance would be ‘reasonable’ unless the technology were sufficiently reliable for such a purpose. The underlying purpose of FRE 703 is recognition that experts rely on (otherwise inadmissible) hearsay in forming their opinion. The output of the machine is not likely to be considered hearsay. See text accompanying notes 123–124.

131 Chow et al., *supra* note 23, at 122 (writing, ‘[w]e strongly caution against the direct translation of our protocol for use as a forensic tool in detecting memories for past events’.)

132 Schacter & Loftus, *supra* note 113 at 121 (internal citations omitted).

133 Stephen J. Anderson et al., *Rewriting the Past: Some Factors Affecting the Variability of Personal Memories*, 14 Applied Cognitive Psychol. 435 (2000). In both younger and older adults, a second recall of an autobiographical memory produced a version with less than 50 per cent of facts identical and new detail being added. Younger adults, when compared to older adults, showed more variation in content and output order. In addition, more recent memories showed greater variation than older ones. Together, these findings suggest a shift from dynamic reconstruction to a more fixed memory that is reproduced or recalled. *Id.*

134 See eg Bernstein & Loftus, *supra* note 29111, at 372–73 (recounting literature on false memories and noting that the challenge in applying laboratory studies to the real world is that in the real world, ‘we do not know who is telling the truth and who is lying or which memories are true and which are false’. Moreover, research focused on groups of memories, an individual reporting a memory, or a single ‘rich false memory’ does not indicate whether a particular memory is true or false. They conclude: ‘In essence, all memory is false to some degree. Memory is inherently a reconstructive process, whereby we piece together the past to form a coherent narrative that becomes our autobiography’.) See also C.J. Brainerd & V.F. Reyna, The Science of False Memory (Oxford Psychology Series eds., 1st ed. 2005); Deborah Davis & Elizabeth F. Loftus, *Inconsistencies Between Law and the Limits of Human Cognition: The Case of Eyewitness Identification*, in Memory and Law, *supra* note 6, at 29; Loftus, *supra* note 29; for a review of memory research (and demeanor evidence) for a legal audience, see Mark W. Bennett, *Unspringing the Witness Memory and Demeanor Trap: What Every Judge and Juror Needs to Know About Cognitive Psychology and Witness Credibility*, 64 Am. U. L.R. 1331 (2015).

135 Daubert, 509 U.S. at 592–93 (1993) (adopting the view of scientific validity based on ‘falsifiability’). Andrea Roth summarizes why surviving a *Daubert* hearing is necessary but not sufficient for providing adequate scrutiny of the basis for the expert’s testimony: ‘[f]or machines offering “expert” evidence on matters beyond the ken of the jury, lawmakers should clarify and modify existing *Daubert* and *Frye* reliability requirements for expert methods to ensure that machine processes are based on reliable methods and implemented in a reliable way. *Daubert-Frye* hearings are a promising means of excluding the most demonstrably unreliable machine sources, but beyond the obvious cases, these hearings do not offer sufficient scrutiny. Judges generally admit such proof so long as validation studies can demonstrate that the machine’s error rate is low and that the principles underlying its methodology are sound. But validation studies are often conducted under idealized conditions, and it is precisely in cases involving less-than-ideal conditions… that expert systems are most often deployed and merit the most scrutiny. Moreover, machine conveyances are often in the form of predictive scores and match statistics, which are harder to falsify through validation against a known baseline’. Andrea Roth, *Machine Testimony*, 126 Yale L.J. 1972, 1981–82. (2017).

136 Roth, *supra* note 135, at 2033–34.

137
*Id.* at 2034.

138
*Id.* (citing Christopher D. Steele & David J. Balding, *Statistical Evaluation of Forensic DNA Profile Evidence*, 1 Ann. Rev. Stat. And Its Application 361, 380 (2014)). Roth criticizes the admission of two expert, proprietary DNA systems that have come to different conclusions in a single case, arguing that the basic *Daubert* and *Frye* reliability tests ‘unless modified to more robustly scrutinize the software, simply do not—on their own—offer the jury enough context to choose the more credible system’. *Id.* at 2035.

139 See text accompanying notes 60–72, *supra*.

140 See Roth, *supra* note 135, at 2035 (writing about competing versions of DNA analysis software: ‘These basic reliability tests, unless modified to more robustly scrutinize the software, simply do not—on their own—off the jury enough context to choose the more credible system’.)

141 Rock v. Arkansas, 483 U.S. 44 (1987); Chambers v. Mississippi, 410 U.S. 284 (1973).

142 See eg United States v. Scheffer, 523 U.S. 303 (1998) (holding that a defendant does not have a right to present polygraph evidence). Justice Thomas, in concurrence, wrote that a ‘fundamental premise of our criminal trial system is that “the jury is the lie detector.” Determining the weight and credibility of witness testimony, therefore, has long been held to be the “part of every case [that] belongs to the jury, who are presumed to be fitted for it by their natural intelligence and their practical knowledge of men and the ways of men””. *Id.* at 313 (Thomas, J. concurring) (in this part of the concurrence, Thomas was joined by Rehnquist, Scalia, and Souter); see also Fisher,, at 571 (‘it is likely that Thomas’s words *do* represent a majority sentiment among judges and practitioners’); see also Wilson, 900 N.Y.S.2d at 640 (rejecting expert testimony on fMRI based lie detection because ‘credibility is matter solely for the jury’ and the expert testimony impinged upon the credibility of the witness); see also State v. Porter, 241 Conn. 57, 118 (1997) (‘[T]he importance of maintaining the role of the jury … justifies the continued exclusion of polygraph evidence … [P]olygraph evidence so directly abrogates the jury’s function that its admission is offensive to our tradition of trial by jury’.); Aetna Life Insurance Co. v. Ward, 147 U.S. 76 (1981); United States v. Barnard, 490 F.2d. 790 (9th Cir. 1973); State v. Williams, 388 A.2d 500, 502–03 (Me. 1978) (noting that ‘[l]ie detector evidence directly and pervasively impinges upon that function which is so uniquely the prerogative of the jury as fact-finder: to decide the credibility of witnesses. The admissibility of lie detector evidence therefore poses the serious danger that a mechanical device, rather than the judgment of the jury, will decide the credibility’.)

143 Scheffer, 523 U.S. at 309–10.

144 See Daniel D. Langleben et al., *Polygraphy and Functional Magnetic Resonance Imaging in Lie Detection: A Controlled Blind Comparison Using the Concealed Information Test*, 77 J. Clinical Psychol. 1372 (2016) (a blind, controlled, within-subjects study comparing the accuracy of fMRI and polygraphy in the detection of intentionally concealed information, wherein subjects were questioned about a number they selected using the CIT paradigm and experts made determinations of the concealed information using polygraphy and fMRI data, reporting that fMRI experts were 24 per cent more likely to detect the concealed number than polygraphy experts).

145 While in principle no overt response is required, as in the BEOS procedure, more research would be needed to examine the efficacy of a purely passive viewing (or listening) task procedure. Most of the brain-based memory detection studies published to date have asked participants to make a button-press response to each stimulus.

146 See Rock, 483 U.S. at 61 (vacating an Arkansas rule that categorically excluded hypnotically refreshed testimony because it prevented a criminal defendant from testifying on her own behalf. Chambers, 410 U.S. at 297–98 (rejecting Mississippi’s argument that its ‘voucher rule’ did not violate Confrontation Clause because the petitioner was unable to cross-examine and establish a defense on a witness who accused him of the crime); Marc Jonathan Blitz, Searching Minds by Scanning Brains: Neuroscience Technology and Constitutional Privacy Protection (2017); Kiel Brennan-Marquez, *A Modest Defense of Mind Reading*, 15 Yale J. L. & Tech. 214, 218 (2013) (arguing that a perfected mind reading device would not result in “testimonial” evidence under the Fifth Amendment); Nita A. Farahany, *Incriminating Thoughts*, 64 Stan. L. Rev. 351 (2012) (recommending that society adopts protections to safeguard liberties as neuroscience advances); Farahany, *supra* note 123 (applying Fourth Amendment concerns to evidence); Matthew B. Holloway, Note, *One Image, One Thousand Incriminating Words: Images of Brain Activity and the Privilege Against Self-Incrimination*, 27 Temp. J. Sci. Tech. & Envl. L. 141, 144 (2008) (arguing that the Fifth Amendment protects suspects from being compelled to undergo brain scans to produce fMRI data).

147 In terms of pragmatic analysis, the cost of fMRI scanning is relevant. A typical scanning session lasts 1 hour, and most university imaging centers charge $400–600 per hour. It is unclear whether prosecutors and defense lawyers would contract with universities to buy time on the scanner (and presumably hire an fMRI expert to run the scan session and analyze the data), or whether there would be dedicated state forensic labs or even for-profit companies with MRI scanners to meet the demand. The high cost would mean that public defenders would not likely be able to routinely access fMRI memory detection scans for their clients, but wealthier defendants may have access to such experts and technology.

148 Credibility, Black’s Law Dictionary (10^th^ ed. 2014) (defined as ‘worthiness of belief’).

149 Julia Simon-Kerr makes a compelling case that a witness’s credibility, or worthiness of belief, is a social construct based on ‘his or her culturally-recognized moral integrity or honor. In other words, people are worthy of belief because they comply with norms of worthiness’. Julia Simon-Kerr, *Credibility by Proxy*, 85 Geo. Wash. L.R. 152, 155 (2017). The definition of credibility we employ in assessing ‘machine credibility’ is not this socially-constructed understanding of credibility, which could only attach to witnesses and not to machine output, but rather that something is worthy of being believed because it is what it purports to be—that is, it is close to a veridical fact.

150 See eg Edmund Morgan, *Hearsay Dangers and the Application of the Hearsay Concept*, 62 Harv. L. Rev. 177 (1948)

151 Roth, *supra* note 135, at 1979 (discussing certain machine conveyances as ‘credibility-dependent proof’).

152
*Id.* at 1977–78. See discussion at notes 182–206, *infra.*

153
*Id.* at 1981–82. See discussion at notes 182–206, *infra*.

154 George Fisher, *The Jury’s Rise as Lie Detector*, 107 Yale L.J. 575 (1997).

155 As an example, consider this recent set of events recounted in the New York Times. See Jim Dwyer, *Witness Accounts in Midtown Attack Show the Power of False Memory*, N.Y. Times, May 14, 2015 (reporting on two witnesses, O’Grady and Khalsa, who independently witnessed a police officer shoot a man in the middle of Eighth Avenue in Manhattan. Immediately afterwards, O’Grady reported to a Times reporter that the wounded man was in flight from the officers when shot. Nearly contemporaneously, Khalsa reported to the Times newsroom that the wounded man was handcuffed when shot. The police released a surveillance videotape 5 hours after the shooting, showing that both were wrong: the wounded man had been chasing an officer onto Eighth Avenue, and was shot by that officer’s partner from behind. Instead of being handcuffed, he was shot while freely swinging a hammer, then lying on the ground with his arms at his side, before being handcuffed. As the paper reported, ‘There is no evidence that the mistaken accounts of either person were malicious or intentionally false’.)

156 See text accompanying notes 132–135, *supra*.

157 See eg Bernstein & Loftus, *supra* note 29111, at 372–73. See also Davis & Loftus, *supra* note 133, at 29; Brainerd & Reyna, *supra* note 134; Loftus, *supra* note 134; for a review of memory research (and demeanor evidence) for a legal audience, see Bennett, *supra* note 134.

158 Meixner, *supra* note 7, at 1454. Rosenfeld also advocates that researchers in the field make this distinction for purposes of advancing admissibility goals: ‘If courts continue to consider credibility assessment evidence inadmissible based the [sic] “jury as the lie detector” standard, the CIT [concealed information test]’s admissibility rests on being able to show the judge that the CIT does not assess whether the witness is telling the truth, but only what he recognizes. In the past, loaded terms like “lie detection” have been features in the titles of CIT experiments. This type of language is likely to mislead courts in the future, and the field would do well to draw a distinction between lie detection and memory detection, as Verschuere et al. do in their book. Bruno Verschuere et al., Memory Detection: Theory and Application of the Concealed Information Test, *supra* note 6.

159 Meixner is not alone in this elision. For example, Meegan’s 2008 review of neuroimaging techniques for memory detection argued that ‘[s]ome have erroneously implied that memory detection tests are actually lie detection tests. As should be clear from the previous review, priming effects, old/new effects, and P300 effects measure recognition rather than deception. Moreover, they can (and should) be measured without dishonest responding’. Meegan, *supra* note 14, at 18. See also Bennett, *supra* note 134.

160 Meixner, *supra* note 7, at 1454; see discussion surrounding *Scheffer*, *supra* note 142. Five justices disagreed with Thomas. In concurrence, Kennedy wrote that ‘the principal opinion overreaches when it rests its holding on the additional ground that the jury’s rule in making credibility determinations is diminished when it hears polygraph evidence. I am in substantial agreement with Justice Stevens’ [dissenting] observation that the argument demeans and mistakes the role and competence of jurors in deciding the factual question of guilt or innocence’. Scheffer, 523 U.S. at 318. Kennedy points out that the rule against permitting the jury to hear ‘a conclusion about the ultimate issue in the trial’ was loosened with Federal Rule of Evidence 704(a). *Id.* at 319.

161 John H. Wigmore, Wigmore on Evidence § 875 (2d ed. 1923).

162 Scheffer, 523 U.S. at 313 (internal citations omitted) (in this part of the opinion, Thomas was joined by Rehnquist, Scalia, and Souter).

163 See discussion in note 142.

164 Fisher, *supra* note 154.

165
*Id.* at 578–79 (writing, ‘[A]lthough the jury does not guarantee accurate lie detecting, it does detect lies in a way that *appears* accurate, or at least in a way that hides the course of any inaccuracy from the public’s gaze. By permitting the jury to resolve credibility conflicts in the black box of the jury room, the criminal justice system can present to the public an “answer”—a single verdict of guilty or not guilty—that resolves all questions of credibility in a way that is largely immune from challenge or review. By making the jury its lie detector, the system protects its own legitimacy’.)

166 Fed. R. Evid 404(b); Fed. R. Evid 413–415.

167 Meixner, *supra* note 7, at 1462.

168
*Id.* at 1473 (noting ‘[e]ven the most optimistic studies that are remotely applicable to the courtroom suggest that individual are just over 60 per cent accurate in credibility assessments. Yet courts continue to hold onto the shaky assumption that the jury is capable of being the sole assessor of credibility. This unsophisticated notion should be put to rest… and the only factor concerning the admissibility of expert testimony related to credibility should be its reliability under *Daubert* or *Frye*’.); see also Max Minzner, *Detecting Lies Using Demeanor, Bias and Context,* 29 Cardozo L. Rev. 2557, 2558 (2008) (writing that ‘[j]udges have generally assumed juries make accurate credibility decisions and believe demeanor is the mechanism for deciding whether a witness is telling the truth…. Starting in the early 1990’s, though, legal academics broke from this consensus based on a series of social science studies demonstrating that the test subjects in laboratory experiments correctly determined when a person was lying only slightly more than half the time’.)

169 Roth, *supra* note 135, at 1990.

170
*Id.* at 1998. (‘Most “expert systems”—programs rending complex analysis based on information fed to it by humans—require inputters to provide case-specific information, and those types of machines might misanalyze events or conditions if fed the wrong inputs…. The potential for error stemming from expert systems’ reliance on the assertions of human inputters is analogous to the potential for error from human experts’ reliance on the assertions of others’.)

171
*Id.* at 1992.

172 See eg Supporting Information, *supra* note 92, at 4–5.

173 See Roth, *supra* note 135, at 1992 (describing a hypothetical situation where an eyewitness states they are ‘damn sure’ that a particular suspect robbed them; if cross-examined in court, the eyewitness would clarify that ‘damn sure’ to them means a certainty of 80 per cent. Without that testimony, a fact finder might associate the term with a higher level of subjective certainty. The same defect lies in machine conveyances: if a supercomputer said it was ‘most likely’ that a death occurred from a particular condition, such a term can create different inferences on the supercomputer’s certainty.); Lawrence H. Tribe, *Trial By Mathematics: Precision and Ritual in the Legal Process*, 84 Harv. L. Rev. 1329, 1330 (1971) (writing that trying to reconcile statistical technology in fact finding processes may distort or destroy ‘important values which that society means to express or to pursue through the conduct of legal trials’.)

174 Anders Elkund et al., *Cluster Failure: Why fMRI Inferences for Spatial Extent Have Inflated False-Positive Rates,* 113 Proceedings Nat’l Acad. Sci. 7900, 7900 (2016) (note that the online version was corrected in an erratum, found in 113 Proceedings Nat’l Acad. Sci. at E4929 (Aug. 16, 2016)). This report led to headlines in the popular press. See eg Bec Crew, *A Bug in fMRI Software Could Invalidate 15 Years of Brain Research,* ScienceAlert (July 6, 2016); Kate Murphy, *Do You Believe in God or is that a Software Glitch?*, N.Y. Times (Aug. 27, 2016), Simon Oxenham, *Thousands of fMRI Brain Studies in Doubt Due to Software Flaws*, New Scientist (Jul. 18, 2016).

175 See Emery N. Brown & Marlene Berhmann, Letter, *Controversy in Statistical Analysis of Functional Magnetic Resonance Imaging Data,* 114 Proc. Nat’l Acad. Sci. E3368 (2017). Functional brain imaging analysis is inherently multidisciplinary. The recent report of the NIH Brain Initiative, *BRAIN 2025*, recognizes this and ‘recommends fostering interdisciplinary collaborations among neuroscientists, physicists, engineers, statisticians, and mathematicians to properly collect, analyze, and interpret the data that result from the development of new neuroscience tools’, including fMRI and EEG. *Id.* at E3369 (citing BRAIN Working Group, *supra* note 76).

176 Roth, *supra* note 135, at 1982.

177 Fed. R. Evid. 403; see Brown & Murphy, *supra* note 5.

178 See eg Darby Aono, Gideon Yaffe & Hedy Kober, *Neuroscientific Evidence in the Courtroom: A Review*, 4 Cogn. Research: Principles and Implications 40 (2019); Nicholas Scurich, *What Do Experimental Simulations Tell Us About the Effect of Neuro/genetic Evidence on Jurors?,* 5 J.L. & Biosci. 204 (2018); Shen et al., *supra* note 5 at 332 (‘Across nearly 30 previous studies, including over 50 unique experiments, the only result researchers can agree upon is that there are “conflicting results”’.); Denise A. Baker et al., *Making Sense of Research on the Neuroimage Bias*, 26 Pub. Understanding Sci. 251, 251, 258 (2015) (reviewing findings on ‘all sides of the neuroimage bias question’ and concluding that ‘when neuroimages do sway judgments, it is only under specific conditions that are not yet well understood; as such, an overarching theory is still out of reach’.); Cayce J Hook & Martha J. Farah, *Look Again: Effects of Brain Images and Mind-brain Dualism on Lay Evaluations of research*, 25 J. Cog. Neurosci. 1397–1405 (2013); Nicholas Schweitzer et al., *Neuroimages As Evidence in a Mens Rea Defense: No Impact*, 17 Psychol., Pub. Pol’y, & L. 357 (2011).

179 See eg Shari Seidman Diamond, *How Jurors Deal with Expert Testimony and How Judges Can Help*, 16 J.L. & Pol’y 47 (2007); David H. Kaye & Jonathan J. Koehler, *Can Jurors Understand Probabilistic Evidence?* 154 J. Royal. Statistical Soc. A 75 (1991).

180 See Daniela Schiller & Elizabeth A. Phelps, *Does Reconsolidation Occur in Humans?*, 5 Frontiers Behav. Neurosci., Article 24 (May 2011), at 1.

181 See Iona D. Scully et al., *Does Reactivation Trigger Episodic Memory Change? A Meta-Analysis*, 142 Neurobiology Learning and Memory 99 (2017).

182 Roth, *supra* note 135, at 1977, 1983. Credibility testing is the next step after an admissibility decision: ‘The purpose of credibility-testing mechanisms is not primarily to exclude unreliable evidence, but to give jurors the context they need to assess the reliability of evidence and come to the best decision’. *Id.* at 2023.

183
*Id.* at 1977.

184
*Id.* at 1988–89.

185 See text accompanying notes 106–111, *supra*.

186 Roth, *supra* note 135, at 1977–78.

187 See text accompanying notes 75–79, *supra*.

188 Roth, *supra* note 135, at 1991.

189
*Id.* at 2026.

190 Jenna Burrell, *How the Machine ‘Thinks’: Understanding Opacity in Machine Learning Algorithms*, Big Data & Society (Jan.-Jun. 2016), at 1–2. The two most relevant types of opacity here are ‘opacity stemming from the current state of affairs where writing (and reading) code is a specialist skill’, and ‘an opacity that stems from the mismatch between mathematical optimization in high-dimensionality characteristic of machine learning and the demands of human-scale reasoning and styles of semantic interpretation’. *Id.* at 2.

191 Supporting Information, *supra* note 92, at 4. RLR was used in subsequent studies from the Rissman group, *supra* notes 89, 96, and 104.

192 Choice of machine learning algorithm for assessment of specific brain functions is an evolving area of study. A recent preprint suggests that deep neural networks outperform linear regression when applied to regional level connectivity, but linear regression is more accurate when applied to system-level brain connectivity. See Bertolero & Bassett (preprint), *supra* note 107. The work by Bertolero & Bassett suggests an even more complex way to apply machine-learning algorithms to memory tasks of recall and recognition, with the potential to understand regional and system-level contributions to discrete subfunctions of autobiographical memory.

193 Peth et al., *supra* note 101, at 167.

194 Zhi Yang et al., *Using fMRI to Decode True Thoughts Independent of Intention to Conceal*, 99 NeuroImage 80, 82 (2014).

195 Burrell, *supra* note 190, at 5.

196
*See* Rissman et al., *supra* note 86, at 9853 (using the classifier’s ‘most “confident” predictions’.)

197 Burrell, *supra* note 190, at 5–8.

198 See Rissman et al., *supra* note 86; Uncapher et al., *supra* note 89; Rissman et al., *supra* note 96; and Chow et al., *supra* note 104; though for some kinds of classifications, the importance maps may be uninterpretable to the extent they do not correspond with brain areas known from other work to be characteristically involved in certain cognitive processes. Even for linear classifiers, importance values suffer from interpretability issues. See Pamela K. Douglas & Ariana Anderson, *Feature Fallacy: Complications with Interpreting Linear Decoding Weights in fMRI, in* Explainable AI: Interpreting, Explaining, and Visualizing Deep Learning 353–-78 (Cham, ed., 2019).

199 Burrell, *supra* note 190, at 5–7 (giving an example of opacity in a neural network classifier, which is applied to problems ‘for which encoding an explicit logic of decision-making functions very poorly’. In contrast, a support vector machine is essentially a form of linear regression, and shares features closer to how humans reason.)

200
*Id.* at 9 (noting that one ‘approach to building more interpretable classifiers is to implement an end-user facing component to provide not only the classification outcome, but also exposing some of the logic of this classification’.).

201 For example, courts could attempt to explain to jurors the core details of machine-learning based conclusions in a similar fashion to how eyewitness testimony is currently explained. See discussion on the New Jersey Supreme Court’s attempt at doing so, *supra* note 13.

202 Roth, *supra* note 135, at 2027–29.

203 Elizabeth A. Holm, *In Defense of the Black Box*, 364 Sci. 26 (Apr. 2019).

204 See Curtis Karnow, *The Opinion of Machines,* 19 Colum. Sci. & Tech. L.R. 136, 166–70 (2017).

205 See text accompanying notes 60–72, *supra.*

206 Decisional accuracy is the touchstone for Meixner and for the coherence of Roth’s taxonomy of machine credibility. Although it is not the only value in a jury system, it is probably the most important, as inaccurate verdicts, or the widespread perception of tolerance of inaccurate verdicts, risks undermining the legitimacy of the system. But elsewhere Roth writes about the pressure that automation of trial-relevant decision-making puts on the ‘soft’ systemic values of ‘dignity, equity, and mercy’. Andrea Roth, *Trial by Machine*, 104 Geo L.J. 1245, 1282–90 (2016).

207 Fisher, *supra* note 154, at 705 (writing that juries inherently are a ‘reliable source of systemic legitimacy. …. Moreover, whether by tradition or conscious design, the jury’s verdict has been largely impenetrable. There never has been a mechanism by which the defendant or anyone outside the system could command the jury to reveal its decision making processes. The jury’s secrecy is an aid to legitimacy, for the privacy of the jury box shrouds the shortcomings of its methods’.)

208 State v. Lyon, 744 P.2d 231, 238 (Or. 1987) (Linde, J., concurring).

209 Kiel Brennan-Marquez, *“Plausible Cause”: Explanatory Standards in the Age of Powerful Machines*, 70 Vand. L. Rev. 1249 (2017); see also Jennifer L. Mnookin, *Repeat Play Evidence: Jack Weinstein, “Pedagogical Devices”, Technology, and Evidence*, 64 DePaul L. Rev. 571, 572, 577–78 (2015) (proposing that computer animations/simulations be ‘cross-examined’ prior to admissibility decisions to permit testing of alternative assumptions).

210 James R. McCall, *The Personhood Argument Against Polygraph Evidence, Or “Even If the Polygraph Really Works, Will Courts Admit the Results?”*, 49 Hastings L.J. 925, 942 (1998).

211 Using Fourth Amendment ‘suspicion decisions’ and policing as an object of study, he essentially argues that judges, ‘as supervisors of state power’, require the context-sensitivity that is enabled by explanations to make their decisions. Brennan-Marquez, *supra* note 209 at 1256. This is the fundamental reason to resist automation (that is, the machine-learning enablement) of suspicion decisions, even if they are statistically more accurate. Brennan-Marquez makes a more general point that ‘[e]xplanations matter—and explanatory standards ought to be preserved in the age of powerful machines –because they enable consideration of two sets of values beyond accuracy’. The first is constitutional constraints, and the second is due process, ‘at some level suffused throughout legal decision making, which separates lawful uses of state power from ultra vires conduct…. Accuracy is not the-all and end-all of sound decision making. This does not mean that accuracy is irrelevant. It is certainly *a* value we care about. But it is not the *only* value that we care about. Other values matter. And explanatory standards allow conflict between divergent values to be managed’. *Id.* at 1280–81.

212 Fed. R. Evid 606(b).

213 Andrew Selbst & Solon Barocas, *The Intuitive Appeal of Explainable Machines*, 87 Fordham L. Rev. 1085, 1117–18 (2018).

214 Fed. R. Evid 606(b).

215 Selbst & Barocas, *supra* note 213, at 1118–19 (citing Tom R. Tyler, *What Is Procedural Justice? Criteria Used by Citizens to Assess the Fairness of Procedures*, 22 Law & Soc’y Rev. 103 (1988)); Tom R. Tyler, *Procedural Justice, Legitimacy, and the Effective Rule of Law*, 30 Crime & Just. 283 (2003); Tom R. Tyler, Why People Obey the Law (revised ed. 2006).

216 Tyler*, supra* note 215.

217
*Cf.* McCall, *supra* note 210, at 943.

218 For example, dementia affects an individual’s perspective on their own personal dignity. See *How Dementia Affects Personal Dignity: A Qualitative Study on the Perspective of Individuals With Mild to Moderate Dementia*, 71 J. Gerontology 491 (2016).

219 Simon-Kerr, *supra* note 149, at 161–66.

220 See *Id.* at 186 (nothing that the ‘link between credibility, reputation, and criminality drawn in today’s impeachment rules thus continues to reflect the notion that the indicia of being a bad person, however defined, is also the indicia of a liar’.)

221
*Id*. at 189–91.

222
*Id.* at 158.

223 Justice Linde wrote, ‘One of these implicit values surely is to see that parties and the witnesses are treated as persons to be believed or disbelieved by their peers rather than as electrochemical systems to be certified as truthful or mendacious by a machine…. A machine improved to detect uncertainty, faulty memory, or an overactive imagination would leave little need for live testimony and cross-examination before a human tribunal; an affidavit with the certificate of a polygraph operator attached would suffice. There would be no point to solemn oaths under threat of punishment for perjury if belief is placed not in the witness but in the machine’. Lyon, 744 P.2d at 240 (Linde, J. concurring).

224 Julie Seaman, *Black Boxes: fMRI Lie Detection and the Role of the Jury*, 42 Akron L. Rev. 931, 936–37 (2009).

225 Giridharadas, *supra* note 2.

226 Documents received from C.R. Mukundan (2009) (on file with ERDM) indicate that the Forensic Sciences Laboratory (FSL) of Gandhinagar (in Gujrat) conducted a total of 174 BEOS tests between Sept. 2009 and Dec. 2009. The FSL of Maharashtra (in Mumbai) conducted a total of 108 BEOS tests between 2007 and May 2009.

227 Saini, *supra* note 3.

228 Interview with C.R. Mukundan, in Bangalore, India (Aug. 11–12, 2009) (transcript and notes on file with ERDM). See C.R. Mukundan, Brain Experience: Neuroexperiential Perspectives of Brain-Mind 190 (2007). Mukundan writes, ‘Autobiographic remembrance is a recall of experiences, which may be composed of awareness of experiences consisting of sensations, proprioceptive sensations actions, emotions, and visual and other forms mental imageries’.) *Id.* at 202. See also D.A. Puranik et al., *Brain Signature Profiling in India: It[]s Status as an Aid in Investigation and as Corroborative Evidence—As Seen From Judgments*, *in* Proceedings of All India Forensic Sci. Conference 815 (2009) (paper presented among others during a conference on Nov. 15–17, 2009, in Jaipur, India); see also Saini, *supra* note 3.

229 Axxonet has funded the research and development into BEOS. Interviews with C.R. Mukundan and colleagues in in Bangalore**,** India (Aug. 11–12, 2009) (transcript and notes on file with ERDM); see also Dr Mukundan’s work on the underlying psychological theories of BEOS, detailed in C.R. Mukundan, *Neural Correlates of Experience*, in Health Psychology 46 (S. Agarwala et al. eds., 2009), and his 2007 book, Brain Experience, *supra* note 228.

230 With respect to the P300 related techniques, BEOS was and is not the only brain-based forensic memory detection technology in use in India. Variously called ‘brain mapping’, ‘brain fingerprinting’, and the ‘Brain Electrical Activation Profile (BEAP)’, the other version of brain-based tests derives from work by Lawrence Farwell on P300 event-related potentials. Interviews with Dr M.S. Rao, Director and Chief Forensic Scientist, Directorate of Forensic Sciences, in New Delhi, India (July 30, 2009) (notes on file with ERDM). Documents received from C.R. Mukundan (2009) (on file with ERDM) indicate that the Forensic Sciences Laboratory (FSL) of Karnataka (in Bangalore) conducted 1131 ‘brain mapping’ or ‘P300’ tests between 2000 and 2009. According to a May 13, 2008 letter from India’s chief forensic scientist, Dr M.S. Rao, to the director of the National Institute of Mental Health and Neuro-Sciences, the Karnataka lab refused to participate in meetings with other FSL directors from other states, ‘stating that their findings are still being evaluated in Germany and U.S.’ Dr Rao also mentioned in that letter that the scientific review committee was ‘not at all satisfied by the available P300 technique at Karnataka’.

231 M. Raghava, Director *of Forensic Sciences Not to Accept Panel’s Findings on Brain Mapping,* Hindu, https://www.thehindu.com/todays-paper/tp-national/tp-karnataka/Directorate-of-Forensic-Sciences-not-to-accept-panelrsquos-findings-on-brain-mapping/article15298701.ece (accessed Mar. 7, 2020). See also M. Raghava, *Stop Using Brain Mapping for Investigation and as Evidence*, Hindu, https://www.thehindu.com/todays-paper/lsquoStop-using-brain-mapping-for-investigation-and-as-evidencersquo/article15297427.ece (accessed Mar. 7, 2020). The panel’s conclusions, as reported in The Hindu, roughly followed a *Daubert*-like analysis, in that ‘the concept on which it was based did not have the support of the scientific community’, ‘rigorous research needed to be done in the area of cognitive processes’, ‘the relevance of these procedures for an Indian setting (for example, the influence of various languages) needs to be established’, ‘recording procedures [need to] satisfy optimal standards’, ‘experiments needed to be carried out in standardised [sic] laboratories satisfying established guidelines’, and ‘the operational procedures needed to be uniform across various laboratories, and the explicit criteria for interpretation and report need to be established with valid scientific basis’.

232
*Id.;* see also Letter from Dr. M.S. Rao to Dr. Nagaraia (May 13, 2008) (on file with ERDM).

233 Giridharadas, *supra* note 2. The New York Times article quoted J. Peter Rosenfeld, a psychologist and neuroscientist whose work is extensively reviewed in Part II, as saying ‘Technologies which are neither seriously peer-reviewed nor independently replicated are not, in my opinion, credible. The fact that an advanced and sophisticated democratic society such as India would actually convict persons based on an unproven technology is even more incredible’. *Id.* Neuroscientist Michael Gazzaniga reviewed ‘promotional dossier’ about BEOS and said, ‘Well, the experts all agree. This work is shaky at best’. *Id.*

234 Discussed *supra* note 230.

235 Selvi v. State of Karnataka (2010) AIR 2010 SC 1974 (India). The Court held that such tests could not be involuntarily administered because of the right against self-incrimination in the Indian constitution and concerns about the reliability of the results of compulsorily administered tests. The Court also held that results obtained through the BEAP test, though no overt response is required from subjects, be treated as testimonial evidence, and thus fall within the scope of constitutional Article 20(3)‘s provision against self-incrimination. ‘[T]he compulsory administration of the impugned tests impedes the subject’s right to choose between remaining silent and offering substantive information’. *Selvi* at paragraphs 161, 221, 223. Involuntarily administered test results are inadmissible, as are the evidentiary fruits of mere investigatory, but compulsory, use. *Id*. Further, the Court held that test results of voluntary examinations ‘cannot be admitted as evidence because the subject does not exercise conscious control over the responses during the administration of the test’, but evidentiary fruits of such voluntary tests may be admissible. *Id.* at 223. BEOS was not specifically considered in the *Selvi* case, but the description and reasoning about the BEAP makes it likely that the holding would squarely apply to BEOS.

236 See *Naveen Kumar to Undergo Neuro-Psychological Tests,* Hindu, https://www.thehindu.com/todays-paper/tp-national/tp-karnataka/gauri-murder-naveen-kumar-to-undergo-neuropsychological-tests/article23552024.ece (accessed Mar. 7, 2020) (reporting that a defendant arrested for his participation in a murder had retracted his alleged consent to undergo ‘polygraph and brain electrical oscillations signature profiling (brain mapping)’ at the Forensic Sciences Laboratory in Gandhinagar. In his retraction, he alleged that ‘police had coerced him to give his consent to the magistrate with the promise that they would help him get bail’.). See also *Teenager gets 20 Years in Jail for Rape, Murder of a Minor,* Hindu, https://www.thehindu.com/todays-paper/tp-national/teenager-gets-20-years-in-jail-for-rape-murder-of-minor/article24990618.ece (accessed Mar. 7, 2020) (quoting a state police spokesman as saying ‘It was first-of-its kind case in which [the state of] Haryana Police had got narco analysis test, polygraph and brain mapping test of the accused done from Forensic Sciences Laboratory, Gandhinagar in Gujarat’.) Gujarat is a different Indian state and one of the few that had BEOS laboratories set up, see discussion *supra* note 230.

237 Jinee Lokaneeta, *Creating a Flawed Art of Government: Legal Discourses on Lie Detectors, Brain Scanning, and Narcoanalysis in India*, 14 Law, Culture, and Hum. (2014) at 13.

238 Jinee Lokaneeta, *Why Narco, Brain Scan & Lie Test Should Be Junked*, Times India, New Delhi, https://timesofindia.indiatimes.com/city/delhi/why-narco-brain-scan-lie-test-should-be-junked/articleshow/61211439.cms (accessed Mar. 7, 2020). See also *Id.* at 2 (reporting on the emergence of brain scanning and ‘narcoanalysis’ in India ‘in a context where physical and mental torture is normalized, and more than a thousand custodial deaths occur each year, despite a strong formal regime of laws and powerful judicial pronouncements’.).

239 Interview with C.R. Mukundan, in Bangalore, India (Aug. 11, 2009). See also Lokaneeta, *supra* note 237, at 12 (quoting a former Director of Forensic Science Laboratories in Karnataka: ‘When the public and human rights activists protest that investigating agencies adopt “third degree” methods to extract information from the accused, it is time the agencies took recourse to the scientific methods of investigation described above’.).

240 Saini, *supra* note 3.

241
*See* Lokaneeta, *supra* note 237, at 14–15 (‘[T]he mere invocation of the term science still appears to be adequate to authorize the techniques. This may be the case because faith in scientific techniques as substitutions for torture has been a recurring feature even in the past. As Justice Katju, a Supreme Court justice wrote: “In western countries scientific methods of investigation are used… Hence, in western countries torture is not formally used during investigation and the correct facts can usually be ascertained without resorting to torture”’..

242 For example, Rosenfeld’s CTP was attempted in a mock terrorist scenario. See Meixner & Rosenfeld, *supra* note 108. The challenge for administering a memory detection protocol in a situation to prevent a terrorist attack is, of course, the uncertain nature of the information available to the investigators. Where might an attack take place? A list of cities is a starting point, but what about the myriad number of targets in a given city? Perhaps a target could be narrowed to an airplane, but which flight? How does one choose stimuli to narrow a range of dates or times in stimulus design?

243 The rules governing the admissibility of evidence are, of course, designed to keep unreliable, un-assessable, and unduly prejudicial evidence out of the fact finder’s purview. Wigmore on Evidence §§ 4b, 9, *supra* note 161. Whether judges are less susceptible than juries to being unduly influenced by such evidence is an empirical question. Research on whether judges are able to disregard inadmissible evidence has some bearing on this issue. It is a reasonable hypothesis that judges might be better than jurors at ‘compartmentaliz[ing] admissible evidence from inadmissible evidence’ to influence their decision, for several reasons: better education, superior abilities to perform a difficult cognitive task, legal training and understanding of the purpose of exclusionary rules, and substantial experience making legal decisions. See Andrew J. Wistrich, Chris Guthrie & Jeffrey J. Rachlinski, *Can Judges Ignore Inadmissible Information? The Difficulty of Deliberately Disregarding*, 153 Univ. Penn. L.R. 1251, 1277. See also *Id.* at 1256, note 21–22 (2005), collecting courts and commentators who have argued that judges are better able than jurors to ignore inadmissible evidence, and noting that ‘[j]udges themselves often apply evidentiary rules more loosely in bench trials than in jury trials on the theory that “the judge, a professional experienced in evaluating evidence, may more readily be relied upon to sift and to weigh critically evidence which we fear to entrust to a jury”’. (internal citation omitted). But other courts and commentators are skeptical that judges are better than jurors at disregarding inadmissible evidence, *Id.* at 1257, and ‘[s]till others assert that judges can disregard inadmissible information in some circumstances, but not in others’. *Id*. at 1258. In psychological experiments simulating different types of judicial decisions, the authors found mixed results: ‘some types of highly relevant, but inadmissible, evidence influenced the judges’ decisions. We also found, however, that the judges were able to resist the influence of such information in at least some cases, namely those directly implicating constitutional rights’. *Id.* at 1259. Unsurprisingly, the empirical data on how mock juries react to instructions to deliberately disregard inadmissible evidence is also full of divergent outcomes. *Id.* at 1270–75.

244 Lyon, 744 P.2d at 240 (Linde, J., concurring).

245 An interdisciplinary research team based in New Zealand has completed a pilot phase of testing ‘forensic brainwave analysis technology’, attempting to replicate and extend the work of Lawrence Farwell and Peter Rosenfeld. See Robin Palmer, *Time to Take Brain-Fingerprinting Seriously? A Consideration of International Developments in Forensic Brain Wave Analysis (FBA), In the Context of the Need for Independent Verification of FBA’s Scientific Validity, and the Potential Legal Implications of its Use in New Zealand*, Te Wharenga—N.Z. Crim. L. Rev., 330 (2018). The pilot project was funded by the New Zealand Law Foundation, which announced a terminal funding round of June 2020 for all projects. See *The Law Foundation*, New Zealand Law Foundation*,*  https://www.lawfoundation.org.nz (last visited Apr. 17, 2020, 10:08 AM). In addition to the pilot study, researchers are focusing on the ‘legal, ethical and cultural impacts of FBA testing is a crucial corollary to the attempted scientific validation of the science underpinning forensic brainwave analysis. This is because legal challenges to the admissibility in court of FBA evidence will not be confined to attacks on FBA’s scientific reliability and accuracy: admissibility challenges based on alleged rights violations flowing from the use of FBA technology at both investigation and trial stages are just as likely’. Palmer, *supra* note 245, at 355.

246 Two examples will suffice. First, while polygraphs are excluded as evidence in most jurisdictions (absent stipulation), they are routinely used in investigations. See Nat’l Research Council, *supra* note 24, at 3. Second, roadside drug tests are inadmissible in nearly every jurisdiction, yet are routinely used in the field and accepted as the only evidence in plea deals by prosecutors and judges in numerous jurisdictions. See R. Gabrielson & T. Sanders, *How a $2 Roadside Drug Test Sends Innocent People to Jail*, N.Y. Times Mag. at 9 (joint investigative report with Pro Publica), https://www.nytimes.com/2016/07/10/magazine/how-a-2-roadside-drug-test-sends-innocent-people-to-jail.html (accessed Apr. 17, 2020).

247 In Japan, a physiological (but not brain-based) version of the CIT is used in both investigations and courtrooms. See Akemi Osugi, *Daily Application of the Concealed Information Test, in* Memory Detection: Theory and Application of the Concealed Information Test, *supra* note 6, at 253–75; see also Izumi Matsuda et al., *Broadening the Use of the Concealed Information Test in the Field*, 10 Frontiers Psychiatry, Article 24 (Feb. 2019). Regarding India, see Lyn M. Gaudet, *Brain Fingerprinting, Scientific Evidence, and Daubert: A Cautionary Lesson from India*, 51 Jurimetrics, J.L., Sci., and Tech. 293 (2011).

248 See eg Amanda Pustilnik, *Evidence Without Law*, work in progress (2020), (writing, ‘As the case of the roadside drug test demonstrates, an inadmissible, unreliable form of evidence, which has resulted in potentially thousands of false and faulty convictions, remains law enforcement’s best friend. Inadmissibility based on substantive unreliability is no barrier to securing guilty pleas and closing cases. Nor does inadmissibility of the drug tests appear to bear heavily on defender behavior: Defenders do not refuse to plead on the grounds that the state cannot prove its case with the inadmissible test. Instead, they counsel their clients to plead. There is no reason to think that the dynamic would be otherwise with newer, apparently more sophisticated and reliable forms of technological evidence’.)

249 While claiming no expertise in the complexities of U.S. asylum law, given current events it is worth pointing out that asylum hearings centrally depend upon credibility assessments. To qualify for asylum, a claimant must have a ‘well-founded’ fear of persecution that is the primary motivation for seeking refuge, the persecution must be on account of one of the statutorily specified bases of the refugee definition, and the alien must be unwilling or unable to return to their country of origin because of persecution or a well-founded fear of persecution. Richard D. Steel, Steel on Immigration Law § 8.8 (2018–19 ed.) The testimony of the applicant is sufficient to sustain this burden only if the adjudicator is satisfied that the testimony is credible, persuasive, and refers to specific facts sufficient to demonstrate that the applicant is a refugee. *Id.* In Oct. 2017, Attorney General Jeff Sessions decried the surge in asylum claimants as evidence of ‘rampant abuse and fraud because of unmeritorious claims of fear’. See Jeff Sessions, Attorney General of the USA, *Remarks to the Executive Office for Immigration Review* (accessed Apr. 17, 2020) (transcript of remarks at https://www.justice.gov/opa/speech/attorney-general-jeff-sessions-delivers-remarks-executive-office-immigration-review). Sessions’ claim, of course, fails to account for changes in the base rate—that is, changes in world or country conditions that drastically increase the number of persecuted persons legitimately seeking refugee status.

250 See eg Juliet Cohen, *Errors of Recall and Credibility: Can Omissions and Discrepancies in Successive Statements Reasonably Be Said to Undermine Credibility of Testimony?* 69 Medico-Legal J. 25 (2001) (reviewing research that it is unusual for recall to be accurately reproduced, and that stories change for many reasons that do not necessarily indicate prevarication); Jane Herlihy, Peter Scragg, and Stuart Turner, *Discrepancies In Autobiographical Memories—Implications for the Assessment of Asylum* See*kers: Repeated Interviews Study*, 342 British Med. J. 324 (2002) (reporting that discrepancies in an individual’s accounts were common, more so in individuals with high levels of post-traumatic stress when the length of time between interviews increased, with more discrepancies in peripheral rather than central details).

251 See eg Safiya U. Noble, Algorithms of Oppression: How Search Engines Reinforce Racism (2018); Tal Zarsky, *The Trouble with Algorithmic Decisions: An Analytic Road Map to Examine Efficiency and Fairness in Automated and Opaque Decision Making*, 41 Sci., Tech., Hum. Values 118 (2015); Reuben Binns, *Algorithmic Accountability and Public Reason*, 31 Philo. & Tech. 543 (2017).

252 See Tribe, *supra* note 173, at 1378–93 (describing, in Part II of the piece, the role that mathematics can play in the design of procedural trial rules); see also the efforts of a few state court systems to update trial procedures and jury instructions to systematically account for scientific understanding of eyewitness memory, *supra* note 13.

